# Pressurized DNA state inside herpes capsids—A novel antiviral target

**DOI:** 10.1371/journal.ppat.1008604

**Published:** 2020-07-23

**Authors:** Alberto Brandariz-Nuñez, Scott J. Robinson, Alex Evilevitch

**Affiliations:** 1 Department of Pathobiology, College of Veterinary Medicine, University of Illinois at Urbana-Champaign, Urbana, Illinois, United States of America; 2 Beckman Institute for Advanced Science and Technology, University of Illinois at Urbana-Champaign, Urbana, Illinois, United States of America; 3 Department of Experimental Medical Science, Lund University, Lund, Sweden; LSU Eye Center Department of Ophthalmology, UNITED STATES

## Abstract

Drug resistance in viruses represents one of the major challenges of healthcare. As part of an effort to provide a treatment that avoids the possibility of drug resistance, we discovered a novel mechanism of action (MOA) and specific compounds to treat all nine human herpesviruses and animal herpesviruses. The novel MOA targets the pressurized genome state in a viral capsid, “turns off” capsid pressure, and blocks viral genome ejection into a cell nucleus, preventing viral replication. This work serves as a proof-of-concept to demonstrate the feasibility of a new antiviral target—suppressing pressure-driven viral genome ejection—that is likely impervious to developing drug resistance. This pivotal finding presents a platform for discovery of a new class of broad-spectrum treatments for herpesviruses and other viral infections with genome-pressure-dependent replication. A biophysical approach to antiviral treatment such as this is also a vital strategy to prevent the spread of emerging viruses where vaccine development is challenged by high mutation rates or other evasion mechanisms.

## Author summary

This work presents a proof-of-concept for anti-herpes treatment based on a novel mechanism of action that interferes with ejection of herpes genome into a host cell by perturbing the viral genome pressure inside the virus’ protein shell, termed a capsid. This interference stops viral infection. Targeting the pressurized DNA state inside a herpes capsid presents a platform for development of broad-spectrum treatments for all human–and animal herpesviruses that are not susceptible to mutation-based resistance development.

## Introduction

*Herpesviridae* are a leading cause of human viral disease, second only to influenza and cold viruses[1–3]. The *herpesviridae* family includes a diverse set of viruses, nine of which are human pathogens[4]. Among these, latent herpes simplex type 1 (HSV-1) and herpes simplex type 2 (HSV-2) infections frequently reactivate to result in recurrent acute oral and genital lesions, as well as encephalitis[2]. Others, such as varicella-zoster virus (VZV), can reactivate decades after the initial infection and may result in painful and debilitating recurrences of shingles[5]. Infections with Epstein-Barr virus (EBV) and Kaposi’s sarcoma-associated herpesvirus (KSHV) are associated with oncogenic transformation[6, 7]. One of the most clinically challenging herpes infections is caused by human cytomegalovirus (CMV), a leading cause of birth defects and transplant failures. There are no effective drugs for CMV without significant toxicities (especially during pregnancy, with high risk to the fetus), and resistance to existing anti-CMV drugs is widespread[8]. Herpesvirus infections are lifelong, with latency periods between recurrent reactivations, making treatment difficult. There is a large unmet need for anti-herpes drugs that can reduce the frequency and severity of recurring outbreaks and control viral replication in the immunocompromised[4, 8]. Currently approved herpetic antiviral agents target and disrupt multiple aspects of the viral life cycle, except for DNA release into the cell nucleus[8]. The most common antiviral drugs are nucleic acid analogs that target viral DNA polymerase and interfere with gene replication[8]. Resistance can, however, quickly evolve from even a single amino acid substitution in the targeted protein, which has been the scourge of most antiviral therapeutics[9]. This has led us to search for antiviral targets that do not involve viral proteins. In this work, we discovered that the mechanically stressed and pressurized state of strongly confined viral DNA packaged in a herpesvirus capsid presents an evolutionarily static target for antiviral therapies that is independent of nucleotide sequences. This approach should prevent development of drug resistance because it targets DNA-DNA electrostatic- and steric interactions (as opposed to specific amino acid or nucleotide sequence) that are unique to tightly packaged herpes DNA[10] and absent elsewhere in the host cell. DNA confined in herpesvirus capsids is at the extreme end of the packing scale at 55% by volume, forming a hexagonally ordered structure with surface separations between negatively charged DNA strands of only ~11Å or less[11–15]. In comparison, the chromosomal DNA packing density of a cell is only ~5–10% by volume[16–18].

Herpesviruses consist of a double-stranded (ds) DNA genome packaged within a protein shell, termed the capsid, that is surrounded by an unstructured protein layer, the tegument, and a lipid envelope. After binding at the plasma membrane, the viral lipid envelope is lost, and partially tegumented viral capsids enter the cell cytoplasm and are transported toward the nucleus. The viral capsid ejects its genome upon docking at a nuclear pore complex (NPC), which forms a passageway for molecular traffic into the nucleus[19, 20]. Herpesviruses package their micrometer-long genome into a nanometer-scale capsid. This tight packing results in repulsive electrostatic forces between negatively charged neighboring DNA helices and bending stress on the packaged genome due to the stiffness of its dsDNA. Our laboratory discovered and measured a high internal DNA pressure of 20 atmospheres in HSV-1 capsids, resulting from this genome confinement[21]. Similar capsid pressure is expected in all types of herpesviruses as well as in dsDNA tailed bacteriophages due to high intracapsid dsDNA packing density[22–24]. Earlier we had measured a pressure of 35 atmospheres in phage λ [24], which has higher DNA packing density than that within a HSV-1 capsid.

We recently showed that this pressure powers ejection of the viral genome into a cell nucleus during HSV-1 infection, providing the first demonstration of a pressure-dependent infection mechanism in eukaryotic viruses[25]. In several parallel studies, we also found that addition of polyvalent cations that penetrate the viral capsid (spermine^4+^) can strongly reduce or eliminate intracapsid DNA pressure through modulation of electrostatic, hydration, and steric interactions between packaged DNA strands[14, 26, 27]. In this work we reconcile our observations and propose for the first time using the pressurized DNA state inside the herpesvirus capsid as a novel target for antiviral agents that can interfere with viral DNA ejection into a cell nucleus. We identified compounds (small polycationic molecules with molecular weights of <800 Da) that are able to permeate the herpesvirus lipid envelope and capsid pores, bind to intracapsid DNA, and condense the packaged genome, thereby removing the capsid genome pressure. We show that this halts viral DNA ejection into a nucleus. The compounds are polyamines that have been previously shown to strongly condense dsDNA[28–30]. Condensation of DNA in a herpes capsid occurs through screening of DNA-DNA net repulsive interactions and introduction of net attractive interactions between the neighboring DNA strands, thus acting as an electrostatic and steric DNA-DNA “zipper”[30–34]. We selected compounds that are commercially available but not previously used to treat herpesviruses, providing an attractive opportunity for drug repurposing[28–30, 35]. Each selected compound is from a different class of main DNA-condensing polyamines: a polypropyleneimine [PPI] dendrimer, a branched polyethyleneimine, and a linear polypeptide. In parallel, we show that these compounds have low cytotoxicity. This suggests that they can serve as lead compounds for further anti-herpes drug development and present a new class of broad-spectrum treatments for all nine human herpesviruses as well as animal herpesviruses (with animal viruses we refer here to veterinary use). Several of the compound classes we selected have been tested in vivo for gene therapy applications; detailed information is available on their pharmacology, formulation, dose, and potential toxicity[28–30, 36–40]. It is important to emphasize that due to the polycationic nature of these polyamine compounds, their permeability through the cell membrane is limited, as shown below. The scope of this work, however, is a proof-of-concept showing that small-molecule polyamine compounds, at concentrations that are not toxic in vitro and in vivo (also verified in this work), can successfully target pressurized DNA state in a herpesvirus capsid bound at the nuclear pore complex and block viral DNA ejection into a host nucleus. This subsequently prevents infection. In order to demonstrate this mechanism of action (MOA) and avoid the issue of limited compound cell penetration, we use a reconstituted isolated nucleus system that accurately reproduces capsid-nuclei binding and nuclear transport of the herpes genome into living cells, which leads to infection[41–44] (the term “infection” denotes the introduction of viral nucleic acid into a host cell by a virus[45]). Drug delivery, including cell membrane permeability, is a challenge in the development of most antiviral agents (e.g. herpesvirus nucleoside drugs, such as penciclovir, had to be modified into a prodrug due to poor solubility in water and as a result poor oral bioavailability[46]). We are currently developing a strategy for efficient delivery of the lead polyamine compound into cells[47]. This will facilitate a demonstration of the MOA in infected cells.

This paper is divided into two sections. In *Section 1*, using solution small angle X-ray scattering (SAXS), we selected small polyamine molecules capable of permeating herpes virions and “turning off” the viral DNA capsid pressure. Our selection criteria were based primarily on the compounds’ ability to condense dsDNA to a tighter packaging density than that found in a herpes capsid under physiologic conditions (ensuring that encapsidated DNA is no longer stressed and does not exert pressure on the capsid walls), with the small molecule size allowing capsid pore permeability and low toxicity. The cell toxicity of the various compounds was tested with standard in vitro proliferating cell culture assays[48]. In vivo toxicity was tested in mice (data shown in Supporting Materials). The toxicity studies were conducted under an NIH/NIAID preclinical service agreement.

In *Section 2*, we developed a MOA-assay for testing selected compounds that focuses on the step where herpes capsids bind to NPCs on the nuclear membrane and eject viral DNA into nuclei[48]. Using a reconstituted isolated nuclei system[41], visualized with ultrathin-sectioning transmission electron microscopy (TEM or EM) and super resolution fluorescence microscopy, we demonstrated that the compounds prevent pressure-driven DNA ejection during the herpesvirus infectious cycle.

These combined assays and the proposed mechanism of action constitute a drug discovery approach for the development of novel broad-spectrum antiviral compounds that treat herpesviruses as well as other resistant and emerging viral infections. This is a biophysical approach to treat viral infections independent of the type of virus within the same virus family. This is a vital strategy for treatment of viruses with high mutation rates or other evading strategies that pose a challenge for vaccine development.

## Results

Although our broad-spectrum MOA is expected to block DNA ejection in all herpesviruses, we chose HSV-1 as a prototypical experimental system for herpesviruses, due to the ease of growing and purifying large quantities of virions and capsids[49].

First, we identified selection criteria for antiviral compounds. To reach the encapsidated viral DNA, the compounds need to permeate the lipid envelope around the capsid, then permeate the capsid wall, and finally condense the DNA in the capsid, inhibiting its ejection. Cryo-EM reconstructions have shown that the axial channels through the HSV-1 capsid are ~20Å in diameter (pore size can vary between types of herpesviruses)[50, 51]. To fit through this channel, compounds must not exceed the molecular weight (MW) of 4000 Da. Compounds that condense DNA are polycationic, with a net positive charge of 3+ or greater, and have a size and shape that permit binding to major or minor DNA grooves and allow them to fit between neighboring DNA strands. This DNA-to-compound binding replaces repulsive DNA-DNA interstrand interactions with attractive interstrand interaction, leading to DNA condensation[21, 31, 52, 53]. Based on the criteria above, we made a comprehensive analysis of DNA condensing agents and selected compounds that ranged from compounds used for gene therapy[28–30, 36, 37] to naturally occurring compounds that condense DNA in sperm cells[30, 32, 54]. The resulting list of approximately 35 DNA condensing molecules with MW cutoff sufficient for permeation of capsid pores included linear polyamines, branched polyethyleneimines, cationic dendrimers, inorganic cationic metal complexes, and cationic surfactants[28, 29, 34, 36, 37, 55–59]. We further refined the compound selection by testing each of these compounds for cell toxicity in proliferating cells under an NIH/NIAID preclinical service agreement (conducted in Mark Prichard’s laboratory, University of Alabama at Birmingham, see details in Materials and Methods). In parallel, using SAXS, we systematically determined average DNA-DNA distance for dsDNA condensed by each compound to verify if the extend of DNA condensation is sufficient to remove the intracapsid DNA pressure. The description of SAXS assays and cell toxicity data is provided below. These quantifications allowed us to determine which compounds remove the intracapsid DNA pressure through condensation of packaged genome at concentrations that are not toxic to cells. Of the molecules we screened, the compounds that showed low cell toxicity and fulfilled other selection criteria above (small MW and DNA condensing properties) were three small molecules: *cationic dendrimer* DAB-Am-4 with MW = 316 Da (a polypropyleneimine, PPI G1), *branched polyethylenimine* (bPEI 600) with six surface amine groups with a charge +6 and MW = 533 Da, and *linear polypeptide* Arg^5+^ with MW = 799 Da. The following experimental analysis will therefore focus on these three compounds.

### 1. Small polycationic molecules “turn off” the intracapsid DNA pressure

**Fig 1:**
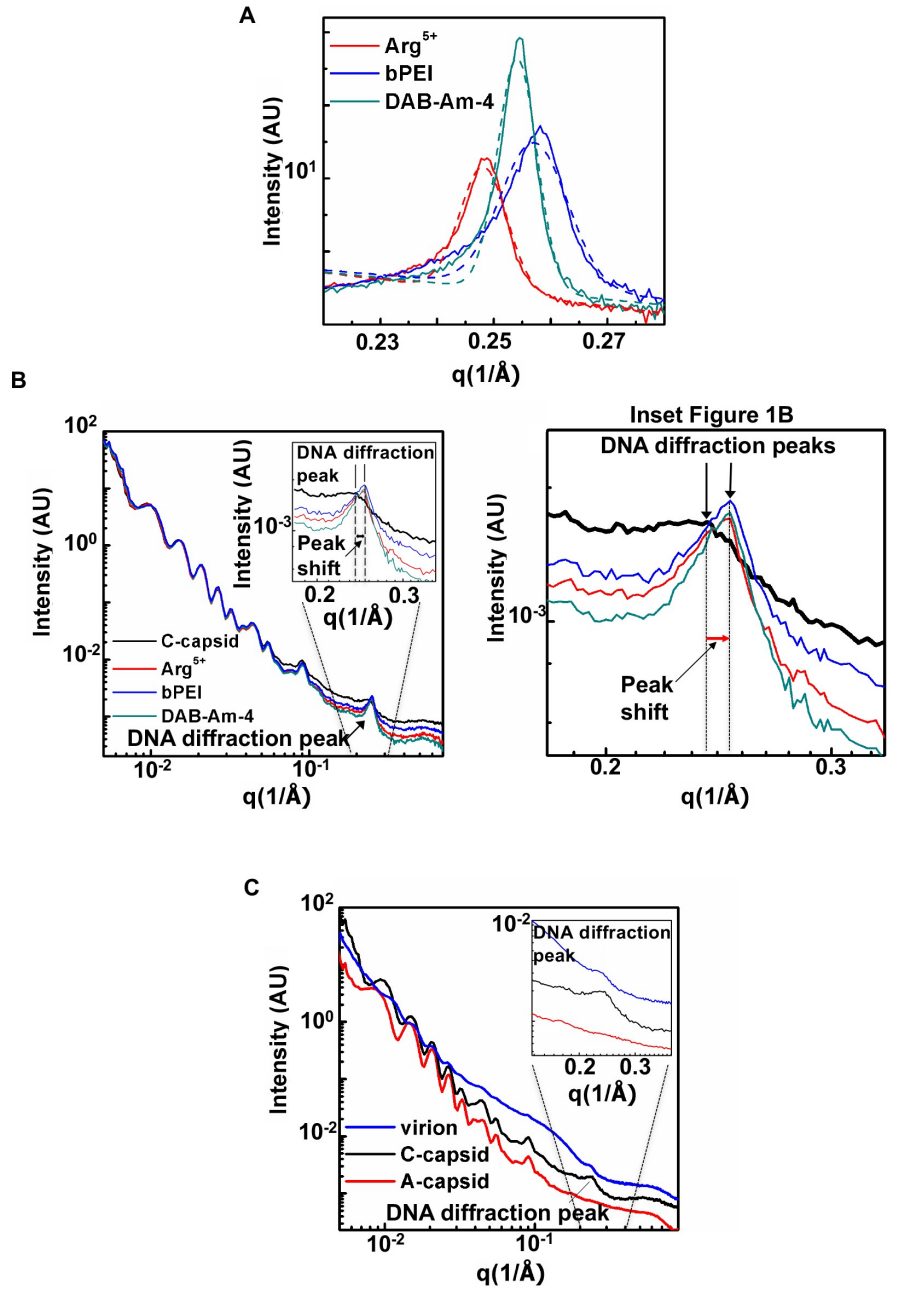
(A) Integrated X-ray scattering intensity, *I*, versus scattering vector *q* for free DNA condensed in TM buffer with added compounds DAB-Am-4, bPEI 600, and Arg^5+^. Compound concentrations corresponded to the N/P ratio of 1.5 required for DNA condensation. Data were collected at 15, 23, and 37°C but are only shown at 37°C (relevant to infection temperature). (B) Integrated X-ray scattering intensity, *I*, versus scattering vector *q* for C-capsids in TM storage buffer without and with added compounds DAB-Am-4, bPEI 600, and Arg^5+^ at 37°C. Compound concentrations corresponded to the N/P ratio of 1.5 required for DNA condensation. The single peak at higher *q* (between 0.2 Å^-1^ to 0.3 Å^-1^) is due to the diffraction from the encapsidated ordered DNA strands. The position of the DNA diffraction peak shifts to higher *q* values when any of the three DNA condensing compounds are added (shown in the Figure inset), indicating that the interaxial distance between packaged DNA strands decreases. (C) SAXS analysis of DNA-DNA spacing in HSV-1 A-capsid, C-capsid, and virion (C-capsid with tegument and lipid envelope). Radially averaged scattered intensity versus the scattering vector *q* for HSV-1 virion (blue curve), C-capsid (black curve), and A-capsid (red curve) at 37 ºC. The inset figure shows a zoomed-in region of the DNA diffraction peak observed for DNA-filled C-capsid and virion. The buffer background was subtracted in (A), (B), and (C). AU, arbitrary units.

**Fig 2:**
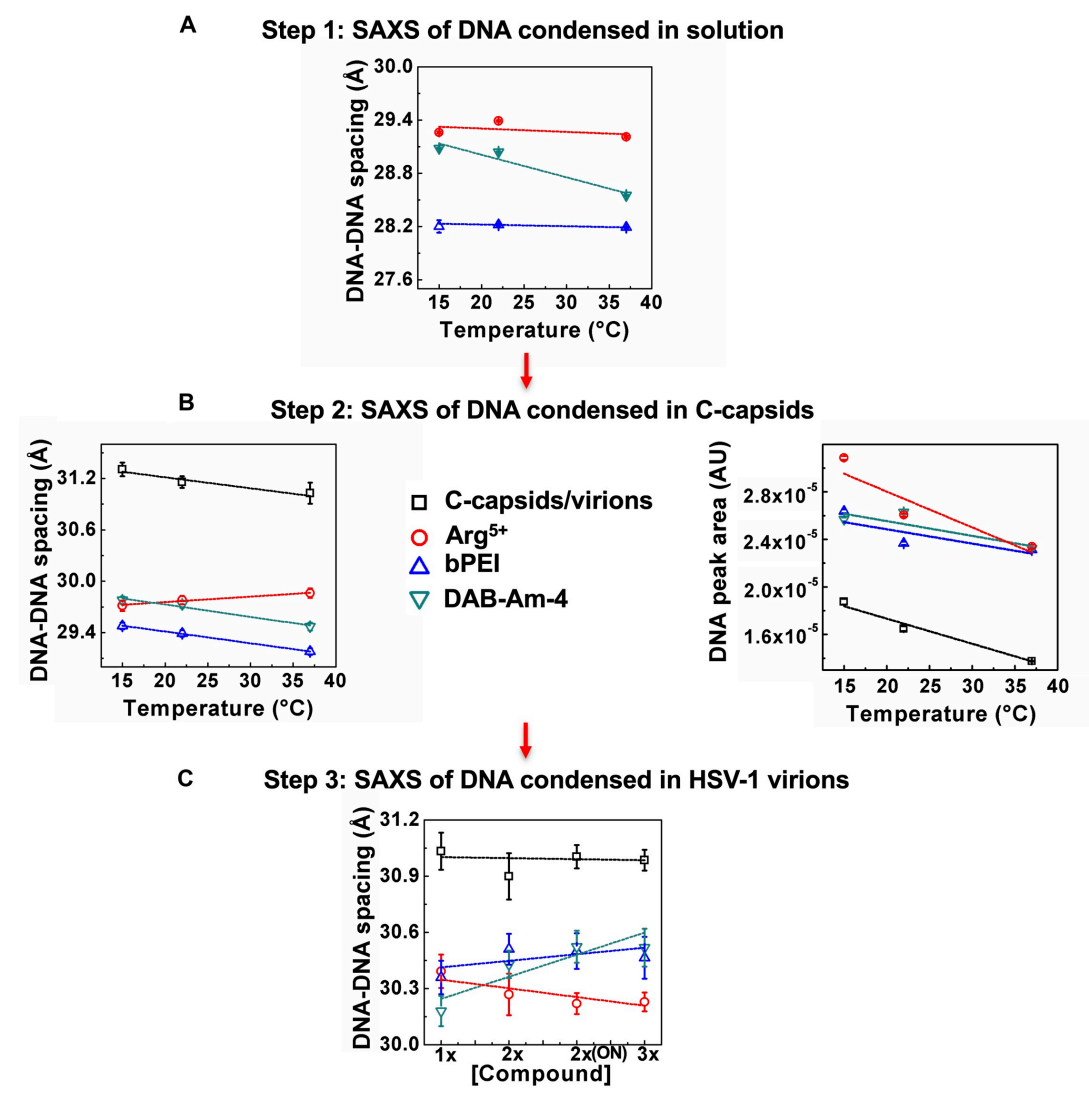
(A) Addition of DAB-Am-4, bPEI 600, or Arg^5+^ compounds condenses free dsDNA in solution, with resulting DNA-DNA interaxial distances, *d*_*s*_, below 31Å (this value is measured for DNA packaged in HSV-1 capsid without compound addition at physiologic conditions). *d*_*s*_ -values were collected at 15, 23, and 37°C and showed minor variation with temperature. (B and C) SAXS screening assay showing that the three selected DNA-condensing compounds mentioned in (A) can penetrate the HSV-1 C-capsid (B) as well as the lipid-membrane-enveloped virions (C) and condense the packaged DNA inside, reducing DNA-DNA interaxial spacing, *d*_*s*_, below 31Å. We tested two- and three-fold higher compound concentration (2x and 3x) than the minimum required for DNA condensation at the N/P ratio of 1.5 used in our study. Incubation time with the compound was 30 min or 12 h (ON: overnight). Temperature and the compound incubation time had only a small effect on DNA-DNA spacing, suggesting that the compound capsid and lipid envelope permeability kinetics are not limited by the diffusion rate. Vertical error bars are from the non-linear fitting of the DNA diffraction peak with a Gaussian function with background subtraction.

In order to demonstrate that our selected DNA condensing compounds permeate the capsid and eliminate intracapsid DNA pressure, we used SAXS to determine the average distance between the centers of tightly packaged, neighboring DNA strands inside the capsid, *d*_*s*_ (DNA-DNA interaxial spacing). Solution SAXS provides direct structural information about the DNA-DNA interaxial spacings determined by the position of the DNA X-ray diffraction peak[11, 60, 61]. The DNA-DNA spacing inside the capsid is determined by repulsive interactions between packaged DNA strands and DNA bending stress, both imposed by confinement inside the capsid wall[14, 62, 63]. To reduce the bending stress, DNA will be pushed towards the capsid walls (providing the lowest curvature), whereas repulsive DNA-DNA interactions will push the DNA strands as far from each other as possible, filling the entire capsid volume and maximizing the *d*_*s*_-value. These energy terms result in a strong DNA pressure on the capsid walls. Although the bending stress term cannot be neglected, the repulsive interstrand interaction term is dominant[14]. In HSV-1 capsids at physiologic conditions, i.e. 37°C in a cell culture or in standard storage TNE-buffer (10 mM TrisHCl, 0.5 M NaCl, 1 mM EDTA) or TM-buffer (50 mM TrisHCl, 10 mM MgCl_2_), the DNA-DNA spacing is *d*_s_* = 31Å[22, 64]. We confirmed this *d*_*s*_-value with SAXS measurements on HSV-1 virions in TM-buffer (mimicking physiologic ionic conditions), as shown in Figs 1 and 2. Thus, in order to “turn off” the intracapsid DNA pressure, the packaged DNA with net DNA-DNA repulsive interactions needs to be condensed with a polycationic compound below the 31Å *d*_*s*_-value. This would indicate that net repulsive DNA-DNA interactions are now replaced with net attractive interstrand interactions. When DNA-DNA spacing in the capsid is no longer maximized, this shows that intracapsid DNA does not “strive” to expand toward the capsid walls and therefore is no longer stressed and pressurized. Furthermore, polycationic DNA condensing molecules strongly decrease dsDNA persistence length, leading to less bending stress[14]. (Persistence length defines the stiffness of a polymer, describing the minimum radius of curvature it can adopt by the available thermal energy. Bending it to a smaller radius requires additional work.) Indeed, it has been previously experimentally demonstrated by us[14, 27] and later by others[65] that when added DNA-condensing polyvalent cations (spermine^4+^) replace the net repulsive DNA-DNA interaction inside a pressurized phage λ capsid with net attractive DNA-DNA interactions, the packaged DNA becomes condensed and jammed, which prevents its ejection from the capsid. It is important to stress that this capsid-pressure-removal criterion (i.e. *d*_*s*_ <31Å) only applies to the addition of DNA condensing compounds because they replace repulsive interactions with attractive interactions between packaged DNA strands. For instance, as we previously showed[21], reducing DNA-DNA spacing in the capsid can also be achieved by external osmolytes that do not permeate the capsid (e.g. polyethylene glycol [PEG]); such external osmolytes generate external osmotic pressure, forcing dehydration of intracapsid DNA with resulting compaction[21, 25]. However, in this case, net repulsive interaction remains, and smaller DNA-DNA spacing only indicates that capsid pressure has been reduced but not necessarily eliminated[21]. It should also be mentioned that cellular polyamines (e.g. spermine and spermidine)[66, 67] can contribute to the weakening of the DNA-DNA repulsions in the capsid[26, 68]. However, most of the polyamines in the cell are bound to the cellular DNA, RNA, and other molecular components[66, 67, 69]. Therefore, the free polyamine concentration in the cell is low and insufficient for condensation of the herpes genome.

To determine if our DNA condensing compounds target pressurized intracapsid DNA in HSV-1 virions, we developed a three-step SAXS assay. In the first step, we confirmed the ability of compounds DAB-Am-4, bPEI 600, and Arg^5+^ to condense the dsDNA in solution. We measured DNA-DNA spacing (*d*_*s*_) in bulk DNA solution in TM-buffer (i.e. free DNA without the capsid), where DNA is condensed by the added compound. This bulk DNA *d*_*s*_-measurement allows for quick prescreening with an in-house SAXS to determine if compounds are capable of binding to dsDNA and reducing DNA-DNA spacing below the physiologic *d*_*s*_-value for DNA packaged in a viral capsid (*d*_s_* = 31Å for HSV-1). Condensed DNA samples showed strong scattering peaks corresponding to interaxial Bragg diffraction from DNA helices packed in a hexagonal array. The Bragg spacing, *d*_*Br*_, and the actual distance between helices, *d*_*s*_, are related by *d*_*s*_ = 2*d*_*Br*_/sqrt(3) (see details in Materials and Methods). Figs 1A and 2A, and S1 Table show that addition of DAB-Am-4, bPEI 600, and Arg^5+^ provided interaxial distances below 31Å (*d*_*s*_ ~28.2–29.4 Å, depending on the compound). As explained above and previously observed[14, 65], this implies that these compounds will yield a net attractive interaction between DNA strands packaged in a HSV-1 capsid, which will suppress intracapsid genome pressure and jam DNA ejection into a cell nucleus during infection. *d*_*s*_-values were collected at 15, 23 and 37°C and showed minor variation with temperature. Compound concentrations were scaled proportionally with DNA concentration in the sample to correspond to the N/P ratio of 1.5, where N provides the number of positively charged amine groups on the polyamine compound, and P provides the number of negatively charged phosphate groups on the DNA. An N/P ratio of ~1–1.5 was previously shown to be sufficient for DNA condensation with polycationic amines[33].

In the second step of the SAXS screening assay, we confirmed that the selected compounds can penetrate the HSV-1 C-capsid (DNA-filled capsid without lipid envelope and tegument proteins) and condense packaged DNA inside. Capsid proteins and packaged DNA have well-resolved SAXS scattering profiles, allowing accurate determination of average DNA-DNA spacing in a capsid[11, 15, 60, 61, 70]. Fig 1B shows integrated X-ray scattering intensity, *I*, versus scattering vector *q* for C-capsids in TM storage buffer without and with added compounds DAB-Am-4 (1.8 mM), bPEI 600 (1.3 mM), and Arg^5+^ (1.5 mM). These compound concentrations corresponded to an N/P ratio of 1.5, required for condensation of DNA packaged in all 10^13^ viral capsids/mL in the sample. This high capsid concentration was needed for high resolution SAXS measurements. Fig 1B and 1C show that in the lower *q* region (0.007Å^-1^ to 0.1Å^-1^), the scattering profile originates from the highly symmetrical icosahedral HSV-1 capsids. The single peak at higher *q* (between 0.2 Å^-1^ and 0.3 Å^-1^) is due to the diffraction from the encapsidated, ordered DNA strands. The short-range DNA-DNA interaxial spacings determine the position of the DNA diffraction peak, whereas the area of this peak indicates how well the DNA strands are aligned relative to each other; that is, it provides information on the total number of ordered DNA base pairs of the encapsidated genome[11, 61]. When DNA inside the capsid becomes less ordered, the DNA peak area decreases (peak becomes wider) as a result of less coherent diffraction. If the genome is completely disordered, the DNA diffraction peak disappears. Analogously to the bulk-DNA SAXS measurements above, the average DNA-DNA interaxial spacing, *d*_*s*_, is calculated using $d=\frac{4\pi }{\sqrt{3}q}$, assuming the hexagonal packing structure of DNA[11]. Forty scans with 1 s X-ray exposure time were collected and averaged for each sample. Vertical error bars for *d*_*s*_-values in Fig 2 are standard deviation values from the non-linear fitting of the DNA diffraction peak with a Gaussian function with background subtraction. SD-values are shown in S1–S3 Tables (see Materials and Methods as well as our previous work for further details[15]). This analysis was applied to all SAXS measurements below. Fig 1B shows that the position of the DNA diffraction peak shifts to higher *q* values when any of the three DNA condensing compounds are added (see the inset in Fig 1B), indicating that the interaxial distance between packaged DNA strands decreases as DNA becomes condensed inside the capsid. Fig 2B and S2 Table show that all three of the selected compounds successfully condense DNA inside the capsid significantly below 31Å (with only a small variation in *d*_*s*_ between 15 and 37°C). Furthermore, Fig 2B shows that the area of the DNA diffraction peak is increased (and the peak becomes sharper) with addition of each of the three compounds between 15 and 37°C, signifying that the DNA becomes more ordered inside the capsid due to compound-induced condensation.

In the third SAXS screening step, we added the selected compounds to whole virions (enveloped capsids) in TM-buffer to verify that the compounds penetrate the lipid envelope, tegument protein layer, and capsid wall. Fig 1C shows integrated X-ray scattering intensity, *I*, versus scattering vector *q* for purified empty A-capsids (A-capsids are essentially identical to C-capsids but without DNA inside[71]), C-capsids (without tegument proteins and lipid envelope), and whole HSV-1 virions. Both C-capsids and virions have a well-resolved X-ray scattering peak corresponding to diffraction from packaged DNA[60] used for *d*_*s*_ determination. S1 Fig shows the SDS-PAGE, verifying protein composition of purified A-, C-capsids, and virions. Fig 2C and S3 Table show that DAB-Am-4, bPEI 600, and Arg^5+^ compounds permeate the virions and condense packaged DNA below 31Å at the physiologically relevant temperature of 37°C. In addition, Fig 2C shows that variation in incubation time from 30 min to 12 h (labeled overnight, ON, in the figure) with compound present, as well as variation in compound concentration (we tested two- and three-fold higher concentration than the minimum required for DNA condensation at the N/P ratio of 1.5 used in our study), have essentially no effect on DNA-DNA interaxial distance, suggesting that compound capsid permeability kinetics is not limited by the diffusion rate of the compound on the timescale of at least 30 min (because selected compounds are much smaller than capsid pores, compound penetration into a capsid likely occurs even faster). During HSV-1 replication, it takes approximately 1 h for HSV-1 to adsorb to a cell surface and an additional 2–4 h for viral capsids to be transported, once fused with a cell membrane, to a cell nucleus where DNA is ejected from capsids. [HSV-1 replication dynamics in cell culture were verified separately and were also verified elsewhere[41]]. Hence, the permeability kinetics of the selected compound occur on a timescale sufficient for compounds to target the DNA inside a capsid. Compound concentrations in this SAXS measurements of virions were the same as in the SAXS measurements of C-capsids above, with virion concentration of 10^13^ pfu/mL.

In order to estimate the relevance of compound concentrations used in the SAXS analysis above for antiviral testing, we conducted cytotoxicity assays for selected compounds. A meaningful value in antiviral drug discovery is the SI_50_ value (*s**elective* *i**ndex*), which is the ratio between *c**ytotoxicity* *c**oncentration* of the compound that reduces cell viability by 50% (CC_50_) and *e**ffective* *c**oncentration* of the compound that reduces viral replication by 50% (EC_50_); that is, SI_50_ = CC_50_ / EC_50_. The desired selective index of at least SI_50_ > 10 shows moderate antiviral compound activity, whereas SI_50_ > 50 shows high antiviral activity. As mentioned above, because selected compounds have limited cell permeability it is not feasible to determine EC_50_ value for antiviral effect. However, as an upper boundary, the EC_50_ value can be set to the compound concentration used in the SAXS analysis above that is required to condense and jam DNA inside all viral capsids (or in virions), making them non-infectious. However, compound concentration in SAXS assays has to be scaled proportionally with viral particle concentration used in a standard in vitro replication plaque reduction assay for EC_50_ value determination[48]. Concentrations of 1.3–1.8 mM for the three compounds above were used for SAXS study to condense DNA in 10^13^ viral particles/mL. For the in vitro replication assay, 10^6^ viral particles/mL are used as a starting concentration for EC_50_ value determination[48]. Therefore, the mM-range of compound concentrations used in the SAXS screening assay correspond to sub-nM (10^−10^ M) EC_50_ compound concentrations, if normalized by the viral particle concentration in viral replication assays.

In parallel, for each DNA condensing compound, we determined the cytotoxicity concentration (CC_50_ value) using standard in vitro cellular replication assays in human foreskin fibroblast (HFF) cells (used as a standard for herpesvirus replication in vitro assays for antiviral compound tests)[48]. Cytotoxicity assays were performed on a parallel set of six-well plates containing confluent monolayers of HFF-1 cells at the same cell amount as used for antiviral plaque reduction assays for EC_50_ value determination, but cells were uninfected[48]. HFF-1 cells showed the following CC_50_ values: 15 μM for DAB-Am-4, 0.03 μM for bPEI 600, and 83 μM for Arg^5+^. We also determined cytotoxicity in the murine MEF cell line, relevant to animal studies. CC_50_ values in MEF cells were: 22 μM for DAB-Am-4 and 0.03 μM for bPEI 600 (Arg^5+^ was not tested). With CC_50_ values in the 10^−8^–10^−5^ M range and estimated upper boundary for EC_50_ values in the 10^−10^ M range for all three tested compounds, the estimated SI_50_ values (SI_50_ = CC_50_ / EC_50_) are 10^2^–10^5^. This suggests that selected compounds should have high antiviral activity, making them promising candidates for further optimization as antiviral agents for herpesvirus treatment. For comparison, using the same cell and virus replication assays (through our NIH/NIAID contract) Acyclovir (used as a control drug) in HFF cells infected with HSV-1 had an SI_50_ ~ 10^2^.

To provide additional demonstration that selected compounds also have low toxicity in vivo, we have tested DAB-Am-4 and bPEI 600 for toxicity in mice through our NIH preclinical service agreement. In this study, three doses of 5, 10, and 25 mg/kg/day (these concentrations are above the estimated EC_50_ values) were administered twice daily via intraperitoneal injection (IP) for 4 days. A control group was treated with PBS. The results of the toxicity study indicated that treatments with bPEI 600 Da and separately with DAB-Am-4 were tolerated well. No signs of distress or significant weight loss were observed during the 4 days of treatment. The results of the laboratory profiles of liver, renal, and blood functions did not show significant differences with the results obtained in the PBS-treated group of control mice (in vivo toxicity data are shown in Supporting Materials).

To summarize, the SAXS observations above show that all three tested compounds bind to intracapsid DNA (as demonstrated by reduced DNA-DNA spacing) and eliminate net repulsive DNA-DNA interactions, replacing them with net attractive interactions condensing DNA in the HSV-1 capsid. This strongly suggests that DNA ejection from capsids into cell nuclei will be inhibited, leading to interference with herpes infection. We confirm this via the MOA-assay described in Section 2 below. It should be noted that although the tested DNA condensing compounds modulate the packaged DNA structure, they had no effect on the empty A-capsid structure, which we also confirmed with SAXS. Thus, the three-step SAXS screening assay can be used successfully for the initial screening of DNA condensing compounds as antiviral agents.

### 2. Reconstituted nuclei system as the MOA-assay for compound activity

Once the capsid pressure was “turned off” through addition of one of the three compounds, it became essential to demonstrate that herpes capsids bound to NPCs do not eject DNA into the cell nucleus. First, we designed an assay that confirmed the proposed MOA. Then we demonstrated that the selected compounds engage with pressurized intracapsid DNA as a target.

To focus on the specific event of herpes DNA injection into a cell nucleus, we built the MOA-assay on previous experiments showing that purified HSV-1 C-capsids bind to NPCs on reconstituted nuclei (isolated from rat liver cells) and eject their DNA into nuclei in the presence of cytosol supplemented with an ATP-regeneration system[25, 41]. We found that tegument-free C-capsids were able to bind efficiently to NPCs and eject DNA into a nucleus[25]. This finding is consistent with a recent study demonstrating that untegumented C-capsids and viral capsids exposing inner tegument proteins on their surface had a similar degree of binding to NPCs[41, 72]. The reconstituted capsid-nuclei system describes the main infection step of viral DNA ejection into a host cell[41–44]. Although the term ‘infection’ usually refers to both viral genome transport into the cell and subsequent replication of the virus, the primary infection in vivo by HSV-1 and HSV-2 is mostly latent; that is, the herpes genome is translocated into the host nucleus without subsequent genome replication[73].

Although we confirmed above with SAXS that DAB-Am-4, bPEI 600, and Arg^5+^ all permeate the lipid envelope of a viral capsid, we found that these polycationic compounds have limited cell membrane permeability and/or difficulty escaping the endosome if they enter the cell through endocytosis[74]. Specifically, we observed that addition of either of DAB-Am-4, bPEI 600, or Arg^5+^ to cell culture, followed by addition of GFP-labeled HSV-1 virions, prevents virus adsorption to the cell (see S2 Fig). This likely occurs due to compounds’ high positive charge leading to electrostatic binding of polycations to negatively charged glycoprotein receptors on the cell membrane, such as heparansulfate proteoglycans (HSPG)[75]. Optimizing compound permeability into cells can be facilitated by e.g. prodrug approach[76], lipid vesicle delivery[47], and nanoparticle delivery[77], as well as other pathways. Compound permeability will be optimized in the future. In this work we show that pressurized state of DNA in a herpesvirus capsid presents a new antiviral target, which is convincingly demonstrated with the reconstituted nuclei system noted below. Furthermore, choosing a reconstituted nuclei system for the MOA-assay (as opposed to infecting a cell culture) provides the benefit of isolating the effect of the compound on the single step of viral DNA ejection while avoiding interference from other processes occurring within the cell during viral replication.

**Fig 3:**
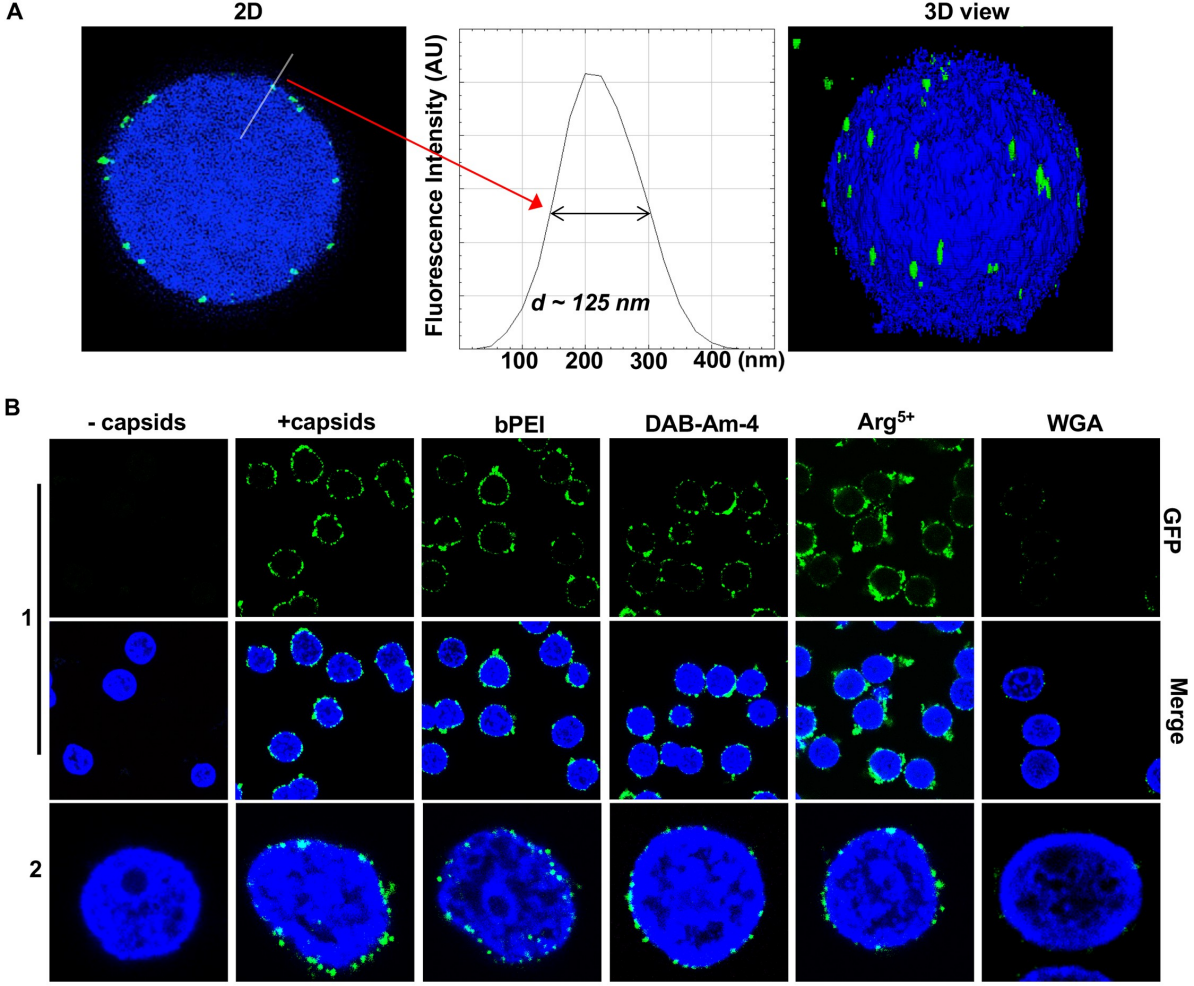
(A) This representative Super-Resolution SIM image shows GFP-HSV-1 C-capsids (green) bound to isolated rat liver nuclei (blue DAPI stain). A histogram of a capsid cross-section profile for a capsid GFP signal along the white line shows that individual C-capsids are resolved. (B) Confocal fluorescence microscopy images show that binding of GFP-HSV-1 C-capsids (green) to DAPI-stained isolated nuclei (blue), in the presence of cytosol supplemented with an ATP-regeneration system, is not inhibited by the addition of selected DNA-condensing compounds at concentrations scaled to correspond to EC_50_ values. The addition of WGA prevents most of the capsid binding to nuclei, which demonstrates that capsids bind specifically to NPCs at the nuclear membrane. The image at the bottom of row 2 is a zoomed-in version of the image of the individual nucleus in row 1.

Fig 3A shows individual GFP-labeled HSV-1 C-capsids (strain K26GFP, the HSV-1 strain expressing GFP-tagged VP26 protein) bound to NPCs on isolated DAPI-stained cell nuclei, imaged using Super-Resolution Structured Illumination Microscopy (SR-SIM)[78]. SR-SIM provides resolutions down to 120 nm, allowing visualization of individual capsids attached to the nucleus. Cytosol was included in the nuclei-capsid mixture to improve capsid binding to nuclei (see Materials and Methods)[72]. In parallel, using confocal fluorescent microscopy (FM), we confirmed that DNA-filled C-capsids bind specifically to the NPCs (as opposed to random binding to the nuclear membrane) and that capsid-NPC binding is not inhibited by the selected compounds, as shown in Fig 3B. As a control, we used WGA (wheat germ agglutinin), which associates with the glycoproteins within the NPC[41, 79] and prevents capsid binding, showing capsid-NPC binding specificity. Prior to testing whether the compound blocks viral DNA ejection into the nucleus (the MOA), we performed additional control experiments with confocal FM and SR-SIM (see details in Materials and Methods). We confirmed that the integrity of the nuclei is not affected by any of the three compounds (DAB-Am-4 at 58.5 μM, bPEI 600 at 39 μM, and Arg^5+^ at 47.3 μM) by showing that fluorescently labeled 70 kDa dextran is excluded from the nuclei interior (S3 Fig). The concentration of each compound provided an N/P ratio of 1.5, required for DNA condensation[33] in all capsids at a C-capsid concentration of ~10^11^ particles/mL. This capsid concentration provides optimal coverage of capsids per nucleus without forming capsid clusters, which would obstruct FM visualization. Compound concentrations in these measurements corresponded to those in the SAXS measurements above, reduced proportionally with the viral particle concentration in this MOA-assay (~10^11^ viral particles/mL for the nuclei-capsid binding assay versus ~10^13^ viral particles/mL in the SAXS assays).

Next we showed that full transport functionality of NPCs is maintained in the isolated nuclei system with compounds present. This was verified with a fluorescently labeled nuclear localization signal (NLS), added to purified rat liver nuclei incubated with cytosolic extracts (as a source of soluble import factors) supplemented with an ATP regeneration system[80], see Supporting Materials for experimental description and S4A Fig. These controls were also reproduced with a digitonin-permeabilized cell assay, which accurately recapitulates capsid-nucleus binding and nuclear import in living cells[42–44]; see Supporting Materials and data shown in S4B Fig. In agreement with observations in the isolated nuclei system (Fig 3B), we also confirmed via a digitonin-permeabilized cell assay that none of the three compounds interfered with capsid-NPC binding in cells. Attachment of GFP-capsids to nuclei was visualized with SR-SIM (S4C Fig). The addition of WGA prevented capsid binding, showing capsid-NPC binding specificity (S4C Fig). Together these findings show that the reconstituted nuclei-capsid system offers a direct MOA-assay for evaluation of the effect of DNA condensing compounds on inhibition of viral DNA injection into a cell nucleus through elimination of genome pressure in the viral capsid.

**Fig 4:**
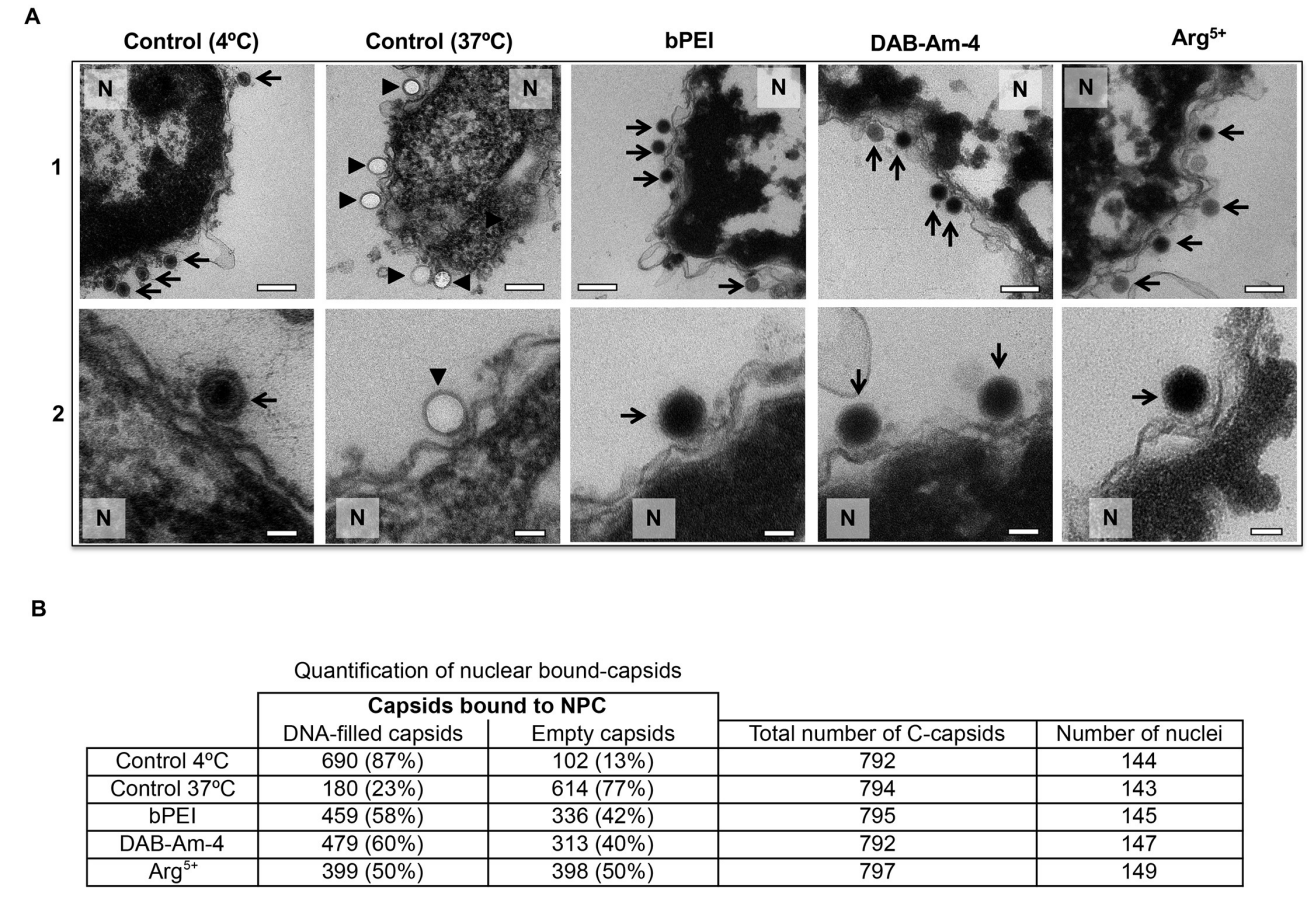
Ultrathin sectioning EM shows that the addition of the selected DNA condensing compounds (DAB-Am-4, bPEI 600, or Arg^5+^) inhibits DNA ejection from HSV-1 C-capsids into a cell nucleus through the NPC. Positive control at 37°C shows complete DNA ejection from C-capsids in the absence of compounds (capsids were mixed with nuclei supplemented with cytosol and ATP-regenerating system). Negative control at 4°C without added compounds or ATP-regenerating system shows that no ejection occurs. In all samples, capsids and nuclei were incubated for 40 min. Bold arrows show empty capsids that ejected DNA, and thin arrows show DNA-filled capsids with DNA condensed inside. **1.** Bar 500 nm. **2.** Bar 90 nm. Representative EM images are shown. At least 790 capsids bound to NPCs were counted for each sample’s statistical analysis, shown in the table below.

Once the integrity and functionality of the reconstituted nuclei-capsid system for MOA testing was established, we applied a stringent test of the antiviral activity of each compound. We used ultrathin-sectioning EM to visualize DNA injection from HSV-1 capsids into isolated nuclei with DAB-Am-4 (58.5 μM), bPEI 600 (39 μM), and Arg^5+^(25 μM). Compound concentrations corresponded to an N/P ratio of 1.5 (scaled proportionally to virus concentration, as described above) (Fig 4). Purified DNA-filled C-capsids were incubated with isolated rat liver nuclei in the presence of cell cytosol supplemented with ATP regeneration system for 40 min at 37°C, providing optimum conditions, found to maximize capsid binding to NPCs with subsequent DNA ejection[41]. Fig 4 shows EM micrographs of capsids attached to NPCs at the nuclear membrane, where ~77% of the docked capsids were empty with fully ejected DNA at 37°C without DNA-condensing compounds. Only capsids that appeared bound to the nuclear membrane are included in the statistics (more than 790 nuclei-bound capsids and over 140 nuclei were counted for each sample analysis, providing good statistics). [Note that micrographs shown in Raw 1 of Fig 4 are lower magnification images (13,500X) which show an area around a nuclear membrane with empty and DNA-filled capsids. For our analysis of capsids bound to NPCs, we only used higher magnification images (25,000X) shown in Raw 2, to identify capsids that were associated with visible NPCs. A larger collection of these EM-micrographs at magnification 25,000X with and without different compounds added is shown in a supporting raw data file (S5 Fig).]

Failure to eject DNA from the remaining ~23% of capsids could be attributed to capsid damage or improper attachment to the NPCs. As shown in Fig 4, when a DNA condensing compound was added to the capsid-nuclei suspension at 37°C in the presence of cell cytosol supplemented with ATP regeneration system, there were ~56% (~50–60% depending on the added compound) that retained unejected DNA. As a negative control, capsids were incubated with isolated nuclei with added cytosol at 4°C for 40 min without ATP regeneration system. As was previously observed[41], these conditions prevented DNA ejection from most viral capsids, with ~87% of HSV-1 capsids retaining their genome (Fig 4). This implies that ~13% of capsids ejected DNA regardless of solution condition, likely due to capsid damage. Thus, the observed ~56% fraction of capsids that retained DNA (or ~44% of capsids with ejected DNA) with added polycation can be normalized with ~13% damaged (empty) capsid fraction, which yields ~64% of capsids with retained DNA. That is a nearly three-fold increase in the fraction of nuclei docked capsids that retained their DNA when polyamine compound was added (compared to only 23% of capsids retaining DNA without compound addition). In parallel, using the fluorescently labeled 70 kDa dextran exclusion assay (described above), we confirmed that capsid binding to NPCs followed by DNA injection into nuclei at these experimental conditions did not affect the integrity of the nuclei.

The fact that not all viral capsids retained DNA with compound addition can in part be attributed to capsid damage with spontaneous viral DNA released outside the nucleus. In S6 Fig using TEM, we observed aggregates of multiple capsids with DNA condensates (from free contaminant herpes DNA) when bPEI was added to a solution of intact C-capsids. The aggregation can be an effect of added polycations acting as a linker between negatively charged DNA and proteins as well as an effect of altered hydrophobic/hydrophilic integrations[81]. This aggregation can induce stress on capsid proteins, leading to capsid rupture and DNA ejection from random locations on the capsid (not necessarily through the capsid portal). Similar capsid aggregation has been previously observed when DNA binding dyes were added to DNA-filled phage capsids, which led to rupture of a small fraction of capsids[81].

It can also be noticed in Fig 4, that cores of DNA-filled capsid appear darker in EM images with added DNA-condensing compounds than without (compared to 4°C negative control sample). This suggests higher DNA density in the capsid core resulting from compound induced DNA condensation. [Denser DNA-filled capsid cores allow fewer electrons to pass resulting in a darker TEM image.] This further indicates that compounds bind to intracapsid DNA and modulate its structure. A collection of TEM micrographs, demonstrating that addition of each of the three DNA-condensing compounds displayed darker capsid core contrast, is shown in a supporting raw data file (S5 Fig).]

**Fig 5:**
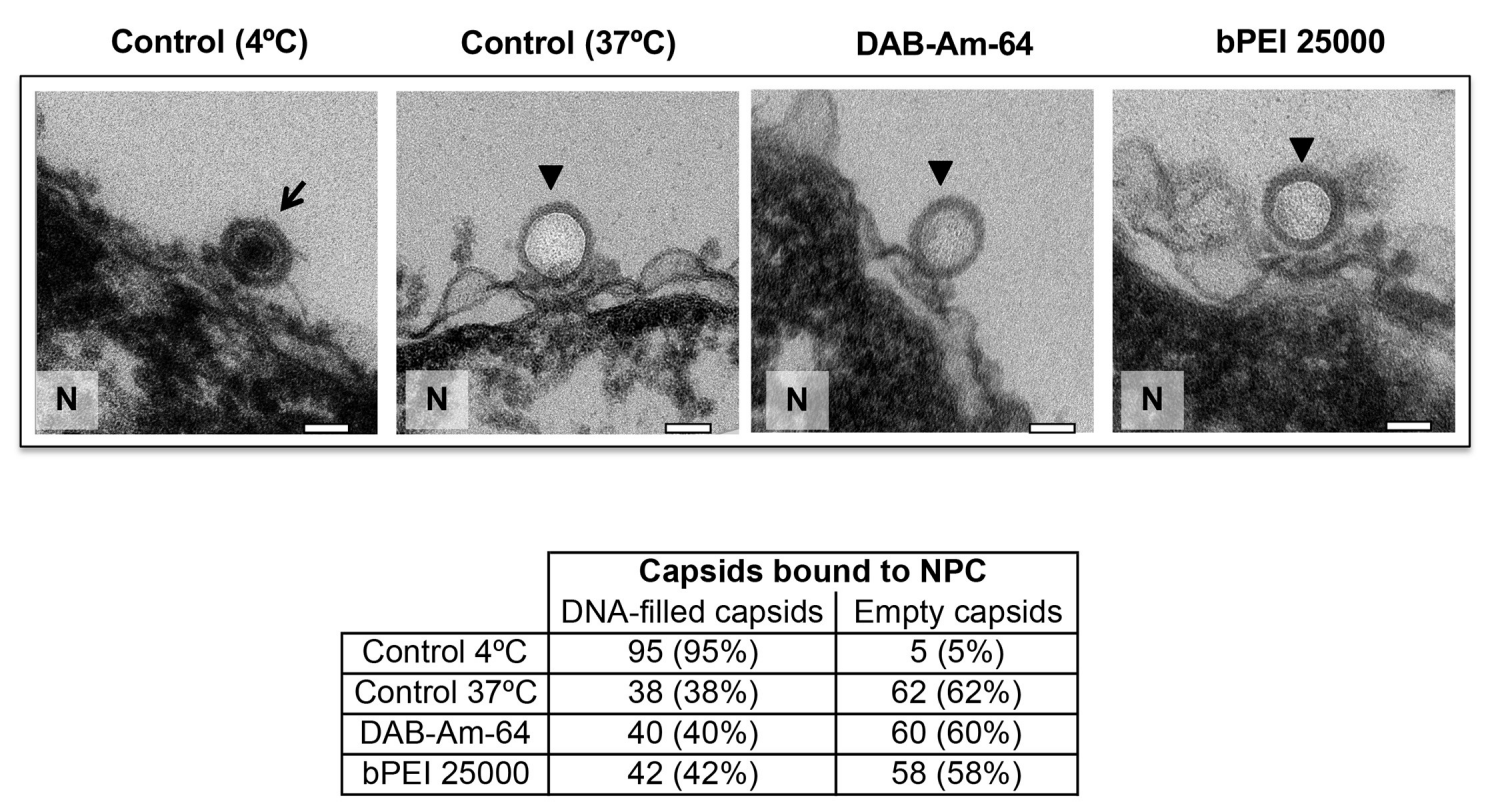
Ultrathin sectioning EM shows that addition of bPEI 25000 and DAB-Am-64 does not inhibit DNA ejection from HSV-1 C-capsids into a cell nucleus through the NPC. Positive control at 37°C shows complete DNA ejection from C-capsids in the absence of compounds (capsids were mixed with nuclei supplemented with cytosol and ATP-regenerating system). Negative control at 4°C without added compounds or ATP-regenerating system shows that no ejection occurs. In all samples, capsids and nuclei were incubated for 40 min. Bold arrows show empty capsids that ejected DNA, and thin arrows show DNA-filled capsids. Bar 90 nm. Representative EM images are shown. At least 100 capsids bound to NPCs were counted for each sample’s statistical analysis, shown in the table below.

In addition, we demonstrate that the observed suppression of viral DNA ejection from capsids into nuclei is caused by the physical interaction between the DNA and the polycation, leading to condensation of the intracapsid genome (turning the capsid pressure off), as opposed to the chemical nature of the compound itself and/or its effect on blocking the NPC channel and interfering with the transport functionality. To show this, we repeated the capsid-nuclei binding experiment above, but this time the small MW compounds bPEI 600 Da and DAB-Am-4 (MW 316 Da) were replaced with the chemically analogous but larger MW molecules bPEI 25,000 Da and DAB-Am-64 (MW 7168.1 Da). It has been previously shown that DAB-Am-64 condenses dsDNA more strongly than DAB-Am-4[82]. Likewise, it was also shown that bPEI 25,000 Da condenses dsDNA more strongly than bPEI 600 Da[83]. The large MW of these compounds, however, prevents them from penetrating the pores in the virus capsid; thus, they cannot condense the viral genome. Using our MOA-assay above (reconstituted nuclei-capsid system), we showed that bPEI 25,000 Da (at 210 nM) and DAB-Am-64 (at 550 nM) did not block ejection of DNA from capsids into reconstituted nuclei, unlike their small MW counterparts; see ultrathin sectioning EM imaging in Fig 5. Compound concentrations corresponded to an N/P ratio of 1.5, as used in all other measurements above. [At least 100 capsids bound to nuclei were analyzed for each sample.] Approximately 38% of nuclei-bound capsids retained their DNA in a control measurement at 37°C without added compounds. The same fraction of nuclei-bound capsids, ~40%, retained their DNA in the capsid with each of the three compounds added at 37°C, demonstrating no inhibition of DNA ejection. In the negative control, at 4°C without an ATP-regenerating system, ~95% of nuclei-bound capsids retained DNA. Similarly, to control measurements above, we confirmed that capsid-binding specificity to the NPC (S7A Fig) and NPC transport functionality (S7B and S7C Fig) in our reconstituted nuclei system are not affected by bPEI 25,000 Da and the DAB-Am-64 addition. This observation further validates the assumption that it is the polycation-induced DNA condensation in the capsid that suppresses DNA ejection through the NPCs by ‘turning off’ the capsid pressure ― not the interference with NPC transport functionality.

These SAXS and EM data clearly demonstrate that DNA ejection from HSV-1 capsids bound to NPCs at cell nuclei is efficiently blocked by DAB-Am-4, bPEI 600, and Arg^5+^ compounds through elimination of intracapsid genome stress that is responsible for DNA ejection. This experimental demonstration provides a foundation for the next step in the drug discovery process, where a lead DNA condensing compound will be optimized for delivery into a cell and then systematically tested for antiherpetic activity with pharmacological in vitro and in vivo assays. Below we discuss additional considerations related to the broad-spectrum use of the proposed MOA and compound binding selectivity for the pressurized intracapsid DNA state.

## Discussion

We provide the first demonstration of a novel MOA for herpesvirus antiviral compound, in which small polycationic molecules prevent viral DNA injection into a cell nucleus by eliminating intracapsid DNA pressure. This in turn blocks viral infection. Our recent measurement of 20 atmospheres of DNA pressure in an HSV-1 capsid[21] was the first demonstration of a pressurized genome state in a eukaryotic virus. This high internal capsid pressure is generated by an ATP-driven packing motor that has been shown to be the strongest molecular motor known[84, 85], located at a unique pentameric capsid vertex. This energetically demanding fit is evolutionarily conserved between orders and families of viruses. Structural features of packing motor components are shared by bacterial and archaeal tailed dsDNA viruses and eukaryotic herpesviruses[86]. This strongly suggests that once DNA is packaged with high force into a capsid, the reverse process of pressure-driven genome release is one of the key mechanisms of viral replication that can be exploited as a novel drug target in different viral systems. Here, we show this for the first time for herpesviruses. High intracapsid DNA packing density resulting in tens of atmospheres of pressure is a distinctive trait of all nine human herpesviruses. We estimated capsid DNA pressures in several types of herpesviruses, using analytical expressions in refs.[62, 87] and EM-measured values for inner capsid diameter (available for VZV, HSV-1, HSV-2, HHV8, and CMV)[22, 23, 88] to compute DNA-DNA electrostatic repulsive force and bending stress. The calculated intracapsid DNA pressures ranged from ~14 atm for VZV to ~46 atm for CMV, which is related to variation in DNA packing density of these viruses[22, 23, 88]. As a reference value to verify the accuracy of calculation, the calculated DNA pressure in HSV-1 was in agreement with our measured value of 19.8 atm[21].

Other types of viruses also exhibit replication steps dependent on the pressurized state of the intracapsid genome. For instance, reoviruses replicate ssRNA to dsRNA inside the capsid during genome packaging; this results in genome packaging densities similar to that of herpesviruses[89]. Such intracapsid replication could be regulated, at least in part, by the internal pressure being generated as newly synthesized dsRNA continues to fill the internal capsid volume, thus increasing genome packing density. Another example is HIV, where, similar to herpesviruses, HIV capsids dock to NPCs at the nucleus and release transcribed dsDNA through the NPC channel[90]. It was recently shown that the reverse transcription process from ssRNA to dsDNA inside the HIV capsid is associated with increasing internal DNA pressure[90]. It has also been suggested that HBV[91, 92] and adenoviruses have pressurized dsDNA[93]. It is plausible, therefore, that the nucleic acid condensing compounds tested here can interfere with pressure-regulated replication and/or suppress viral genome ejection in these viral systems through the proposed MOA.

Besides broad-spectrum treatment, the other most significant advantage of our MOA is the inability of targeted pressurized intracapsid DNA to develop drug resistance. This is achieved by targeting intrinsic physical properties of high negative charge density on the tightly packaged genome, as opposed to specific nucleotide or amino acid sequences that are prone to mutations, which is the case with most current antivirals. However, lowering target binding specificity increases the risk for toxicity by randomly targeting essential cellular components. Although electrostatic interactions between selected polycations and negatively charged DNA are not sequence specific, the hydration and steric interactions that are also responsible for attractive forces between the polycation and packaged DNA strands are highly cooperative and specific to the secondary and tertiary structure of densely packaged DNA inside the capsid[10]. As a result, the attractive energy component leading to DNA condensation is approximately two times larger than the DNA-DNA repulsive-component[10]. This steric specificity of attractive interactions between polyamine cations and packaged herpes dsDNA is not present when polyamine cations interact with other cellular structures (e.g. negatively charged membranes or proteins) through mainly nonspecific electrostatic interactions[10, 30–33]. In addition, the compound binding affinity for the pressurized intracapsid DNA also depends on the negative charge density of the packaged genome. The negative charge density of the intracapsid DNA is significantly higher than that of a cell’s chromosome and acts as an electrostatic “sink” for DNA condensing compounds, where compound binding affinity for chromosomal DNA is potentially lower[30, 32, 94]. This is due to the fact that cellular condensed DNA molecules have about five times lower DNA packing density [DNA density is ~40–50% by volume inside a herpes capsid[22] versus ~10% in the cell chromosomes[30, 32]]. Furthermore, at least 50% of the negative charge of the chromosomal DNA is neutralized by histones[30, 32, 94]; there are no internal capsid proteins neutralizing DNA charges in herpes capsids[84]. It should be noted that dsDNA is also present in mitochondria. However, the mitochondrial membrane is essentially impermeable to external polycationic compounds, allowing the mitochondrion to maintain the transmembrane potential required for ATP synthesis[95]. It can also be mentioned that if these positively charged polyamine compounds will be delivered into a cell cytoplasm through a prodrug or a lipid vesicle approach, the charged polycation of the active compound will likely not be permeating the nuclear membrane (similarly to its inability to permeate the cell membrane), which will also limit targeting of the cellular chromosome.

In conclusion, we have shown for the first time that the pressurized state of the viral packaged genome is a novel target for antiviral therapies. We have identified and tested small-molecule compounds that “turn off” capsid pressure and block viral genome ejection into a cell nucleus, which should subsequently prevent infection. This presents a platform for the development of antiviral treatments with a target that is impervious to resistance and universal to many viruses that afflict humans and animals.

## Materials and methods

### Cells, viruses, and compounds

African green monkey kidney cells (Vero; ATCC CCL-81 from American Type Culture Collection, Rockville, MD) and neonatal human foreskin fibroblasts (HFF-1; ATCC SCRC-1041) were cultured at 37°C in 5% CO2 in Dulbecco’s modified Eagle’s medium (DMEM; Life Technologies) supplemented with 10% fetal bovine serum (FBS; Gibco), 2 mM L-glutamine (Life Technologies), and antibiotics (100 U/ml penicillin and 100 μg/ml streptomycin; Life Technologies). The KOS strain of HSV-1 was used as the wild-type strain. The K26GFP HSV-1 recombinant virus (gift from Dr. Fred Homa, University of Pittsburgh), which carries a GFP tag on the capsid protein VP26, was used in fluorescence studies. The HCMV (human cytomegalovirus) strain, AD169, was obtained from the American Type Culture Collection (ATCC, Manassas, VA). All viruses were amplified on Vero cells or HFF-1 (for HCMV), and titers were determined on either Vero cells or HFF-1 by plaque assay. Viral plaque assays were carried out as follows. Viral stocks were serially diluted in DMEM. Aliquots were plated on six-well trays of Vero cells for 1 h at 37°C. The inoculum was then replaced with 40% (v/v) carboxymethylcellulose in DMEM media. HSV-1 and HCMV plaque assays were incubated for 3 days. The monolayers were stained for 1 h with crystal violet stain (Sigma-Aldrich). After removal of the stain, the trays were rinsed with water and dried, and plaques were counted.

### HSV-1 capsid isolation

African green monkey kidney cells (Vero) were infected with either HSV-1 KOS strain or K26GFP HSV-1 recombinant virus at a MOI of 5 plaque-forming units (PFU) per cell for 20 h at 37°C. Cells were scraped into solution and centrifuged at 3,500 rpm for 10 min in a JLA-16.250 rotor. The cell pellet was resuspended in 20 mM Tris buffer (pH 7.5) on ice for 20 min and lysed by addition of 1.25% (v/v) Triton X-100 (Alfa Aesar) for 10 min on ice. Lysed cells were centrifuged at 2000 rpm for 10 min and the nuclei pellets were resuspended with the addition of 1x protease inhibitor cocktail (Complete; Roche). Nuclei were disrupted by sonication for 30 s followed by treatment with DNaseI (Thermo-Fisher) for 30 min at room temperature. Large debris were cleared by brief centrifugation, and the supernatant was spun in a 20–50% (w/w) sucrose gradient in TNE buffer (500mM NaCl, 10mM Tris, 1mM Na_2_EDTA, pH 8.0) at 24,000 rpm in a SW41 rotor for 1 h. The C-capsid and A-capsid bands were isolated by side puncture, diluted in TNE buffer, and centrifuged at 23,000 rpm for an additional 1 h. Capsids were resuspended in TNE buffer and stored at 4°C. For small angle X-ray scattering (SAXS) studies, C-capsids and A-capsids were dialyzed in TM buffer (10mM Tris pH:7.5, 10mM Mg_2_Cl). To analyze the effect of the polyvalent cationic agents on the viral DNA structure by SAXS, C-capsids were incubated with the compounds at least 4 h before the measurements. The concentrations of the compounds added to induce DNA condensation were calculated in order to obtain a compound charge/bp DNA virus ratio of 1.5. For nuclei-capsid binding studies, C-capsids were dialyzed in CBB buffer (20 mM HEPES-KOH with pH of 7.3, 80 mM K-acetate, 2 mM DTT, 1mM EGTA, 2mM Mg-acetate, 1mM PMSF, and 1X CLAP cocktail).

### Purification of extracellular virions

Vero cells were infected with an HSV-1 KOS strain overnight (21 h at 37°C) at a MOI of 0.1 PFU per cell. Infected cells were scraped into the cell medium, and 5 M NaCl was added to a final concentration of 0.5 M NaCl. Cells were pelleted, and the medium was transferred to SW28 rotor tubes; virions were pelleted out of the medium by centrifugation at 20,000 rpm for 35 min. The resulting pellet was resuspended in 100 μl of PBS plus protease inhibitors. The sample was incubated with DNase I (Thermo-Fisher) at room temperature for 30 min, and then layered on top of a 20–50% sucrose gradient in PBS buffer (10 mM PO4^3−^, 137 mM NaCl, 2.7 mM KCl; SW41 rotor at 24,000 rpm for 1 h). The virion band was collected with a syringe by side puncture of the gradient, transferred to an SW41 tube, diluted 1:5 with PBS buffer, and the virions pelleted. The virions were resuspended in PBS and stored at -80ºC. To analyze the effect of the polyvalent cationic agents on the viral DNA structure by SAXS, HSV-1 virions were incubated with the compounds at different times as described in the figure legends. The concentrations of the compounds added to induce DNA condensation were calculated as described above.

### Gel electrophoresis

Five micrograms of purified extracellular HSV-1 virions, C-capsids, or A-capsids were boiled for 10 min in loading buffer (50 mM Tris-HCl, pH 6.8, 2% SDS, 0.1% bromophenol blue, 10% glycerol, and 2% β-mercaptoethanol) and separated on a NuPAGE 4‒12% Bis-Tris gel (Invitrogen). Protein bands were visualized by Coomassie blue staining.

### SAXS

For bulk DNA measurements with in-house SAXS, concentrated polycation solutions were added to 1 mg/mL free DNA in small increments of concentration. Fibrous DNA samples condensed with tested compounds were centrifuged and the DNA pellets transferred to an X-ray capillary. Condensed DNA samples showed strong scattering peaks corresponding to interaxial Bragg diffraction from DNA helices packed in a hexagonal array. The Bragg spacing, *d*_*Br*_, and the actual distance between helices, *d*_*s*_, are related by *d*_*s*_ = 2*d*_*Br*_/sqrt(3). Typical exposure times were ~ 30 min.

All SAXS measurements of viral capsids were conducted at a concentration of 10^13^ viral particles per mL (the highest technically achievable capsid concentration). Because the capsid or virion volume fraction constitutes only ~1% (unlike concentrated condensed bulk DNA fibers), we had to use a powerful synchrotron X-ray scattering source at the Argonne National Laboratory to produce a sufficient signal-to-noise ratio. SAXS measurements were carried out at the 12-ID B station at the Advanced Photo Source at Argonne National Laboratory. A 12-KeV X-ray beam was used to illuminate the sample with an overall scattering vector q range from 0.006 to 0.850 Å ^−1^. To reduce radiation damage, a flow-cell equipped with a quartz capillary 1.5 mm in diameter was used for running samples. The typical sample volume of DNase-treated C-capsids, A-capsids, or virions in solution (~10^13^ viral particles/ml) was 120 μl and the flow rate was 10 μl/s. Samples were measured in the temperature range of 15–37°C, and the sample temperature was controlled with a Peltier device with ± 0.5°C accuracy. At each temperature, the equilibrium time was 15 min to ensure the solution reached the desired temperature. 40 two-dimensional images were collected for each sample and buffer with X-ray exposure time of 1 s. The two-dimensional scattering images were converted into one-dimensional SAXS data (i.e., intensity versus q) through azimuthal averaging using the software package at the beamline (Igor Pro 7). The one-dimensional SAXS data curves were grouped by sample and averaged, followed by buffer background subtraction. The peaks in the SAXS data below q of 0.1 Å^−1^, for HSV-1 A-capsids, C-capsids, and virions mainly arise from the global shape of the particles, whereas the peak around 0.2 Å^−1^ is the diffraction peak of DNA array. Because the DNA diffraction peak was distorted by the scattering background from the viral particle shape, it was fitted with the summation of a Gaussian curve and a linear background in the q range of 0.16 Å^−1^ to 0.3 Å^−1^; this Gaussian function gave the accurate peak position and area. DNA-DNA interaxial spacing *d*_*s*_ can be calculated from the peak position *q*, i.e., *d*_*s*_ = 4π/sqrt(3)*q*, assuming DNA arrays adopt a hexagonal close packing.

A buffer solution of the dialysis buffer for virus samples was measured using the same SAXS setup, which was further subtracted as the background. After the background subtraction, the scattered intensity *I* versus *q* plot was plotted, and the DNA peak region was truncated. This DNA diffraction peak was fitted with a Gaussian curve plus a linear background using function below, where *q*_0_ is the peak center, *w* is the peak width, *A*_0_ is the peak area, *k* is the slope of the linear background, and *c* is the offset.

$$I=\left(\frac{A_{0}}{w\times \sqrt{\pi /2}}\right)e^{-2{\left(\frac{q-q_{0}}{w}\right)}^{2}}+kq+c$$

The DNA peak area *A*_0_ was chosen as the most convenient measure of the ordered DNA strands because it includes a temperature factor, or the displacement parameter, which signifies the drop in the diffraction peak intensity due to the thermally induced vibration or displacement of the scattering centers.

Vertical error bars for *d*_*s*_-values in Fig 2 show standard deviation values (also provided in S1–S3 Tables). SD-values were obtained from the non-linear fitting of the DNA diffraction peak with a Gaussian function with background subtraction using an Origin software algorithm (we used Gaussian function listed under non-linear fitting tab and added a linear fit to the Gaussian function).

### Rat liver nuclei isolation and cytosol preparation

Nuclei from rat liver cells were isolated, as adapted from previously described protocol[41]. The intactness of nuclei was confirmed by light microscopy, EM (electron microscopy), and FM (fluorescence microscopy) by staining the nuclei with DAPI and by their ability to exclude fluorescently tagged (fluorescein isothiocyanate) 70 kDa dextran. The cytosol was separately prepared using BHK-21 cells. The rat liver samples were a gift from Dr. CheMyong Jay Ko, Department of Comparative Biosciences, University of Illinois at Urbana‒Champaign. A consent to use the sample was obtained.

### Cytosolic extract

The cytosol was separately prepared using BHK-21 cells (ATCC CCL-10). Cells were grown to confluence in 245 x 245 mm plates and collected from the plates by addition of 0.25% trypsin/EDTA (Thermo Fisher Scientific). Collected cells were washed with KEHM buffer (50 mM KCl, 10 mM EGTA, 50 mM HEPES pH 7.4, 2 mM MgCl_2_) and kept on ice. Cells were then resuspended in KEHM buffer supplement with 1 mM DTT and 1x protease inhibitor (complete; Roche) at the volume of 5 mL KEHM per 109 cells. Resuspended cells were broken using an EMBL 8.020 mm cell cracker homogenizer with ball sizes of 8.010, 8.008, or 8.004 nm. To remove the intact cells and nuclei, the sample was centrifugated at 3000 rpm for 15 min at 4ºC. The supernatant was spun down at 80,000 rpm (267,000 g) for 30 min at 4ºC. Only the supernatant was kept as the purified cytosol. The cytosol was frozen in liquid nitrogen and stored at -80ºC for long time-storage.

### Reconstituted nuclei-capsid system

We built an in vitro viral HSV-1 DNA translocation system in which the HSV-1 genome was released into nucleoplasm in a homogenate solution mimicking the cytoplasm environment; see details in previously described protocol in ref.[41]. In a typical system, rat liver cell nuclei were incubated with C-capsids (HSV-1 or GFP-labeled HSV-1) in solution containing: (i) cytosol, (ii) BSA, (iii) ATP regeneration system; see details in ref.[41]. The system was incubated at 37°C for 40 min, sufficient for capsid binding to nuclei. For inhibition studies, wheat germ agglutinin (WGA) was preincubated with the nuclei prior to addition of C-capsids. For compound-induced inhibition of DNA ejection studies, C-capsids were preincubated with the polyvalent cationic agents at the concentrations described in the main text before adding capsids to nuclei.

### Fluorescence microscopy

Vero cell monolayers grown on chamber slides (Lab-Tek) were infected with a GFP-expressing HSV-1(K26GFP) at a MOI of 0.1 PFU per cell. Vero cells were also incubated with the same virus that was previously treated with different concentrations of the compounds to condense the viral DNA. The concentration of the compounds was calculated as described above. At 24-h post infection, the cells were rinsed with PBS three times and fixed by submersion in 4% paraformaldehyde for 15 min. Cells were washed three times with PBS, and 0.5% Triton X100 was added for 5 min to permeabilize the cells. Then the cells were incubated for 5 min with DAPI (4*′*,6*-*diamidino*-*2*-*phenylindole, Thermo Fisher Scientific) to stain the nuclei.

For fluorescence imaging of the reconstituted nuclei-capsid system, GFP-labeled HSV-1 C-capsids were used. After incubation of nuclei-capsids as described above, the buffer system containing purified GFP-labeled C-capsids and nuclei were loaded onto coverslips. Overlay of the confocal 488 (for GFP emitted signal) and 358 (for DAPI emitted signal) channels show the localization of viral capsids onto the nucleus. Images were captured with a Nikon A1R laser-scanning confocal microscope.

### Bacterial expression and purification of GST-NLS-EGFP

Expression and purification of GST-NLS-EGFP in the XL1-Blue bacteria was performed as described previously [96, 97].

### Nuclear import assay

Rat liver cell nuclei were incubated with GST-NLS-EGFP at 37ºC for 40 min in CBB buffer containing: (i) rat liver cell cytosol, (ii) 1 mg/mL BSA, and (iii) ATP regeneration system. The complete binding mixture was loaded onto coverslips and processed for fluorescence imaging as described above. When indicated, nuclei were preincubated with the polyvalent cationic agents at the concentrations described above for 2 h before performing import assays. For WGA treatment, samples were incubated for 15 min at room temperature with transport buffer containing 0.5 mg of WGA/ml before performing import assays.

### Digitonin permeabilization and nuclear import assays

To obtain cytosolic extracts as a source of soluble import factors, Vero cell monolayers (4 x 10^7^ cells) were washed with ice-cold PBS and scraped off the plate into 5 ml of cold PBS. The cell suspension was centrifuged at 600*g* for 10 min at 4°C, and pelleted cells were washed with cytosolic buffer (50 mM HEPES-KOH [pH 7.4], 50 mM KCl, 2 mM MgCl2, 5 mM EGTA, 1 mM DTT, and 1X CLAP cocktail) and resuspended in 0.1 ml of cytosolic buffer. The cell suspension was flash-frozen in liquid nitrogen, and the sample was then placed at 37°C until thawed. The resulting cell lysate was centrifuged at 16,000*g* for 15 min at 4°C in a refrigerated microfuge, and the supernatants were flash-frozen in liquid nitrogen and stored at -80°C. For permeabilization, Vero cells plated on glass slides and grown up to 70% confluence were washed twice with ice-cold CBB buffer and permeabilized with digitonin (25 μg/ml) for 3 min on ice. Cells were then washed twice with ice-cold CBB buffer containing 10 μg of bovine serum albumin/ml. The standard reaction mixtures contained purified import substrates and GST-NLS-EGFP dissolved in complete transport solution. This solution contained CBB buffer supplemented with an ATP regeneration system as a source of energy and cytosolic extracts from Vero cells as a source of soluble import factors. The import reactions were performed for 40 min at 37°C. The samples were then processed for fluorescence imaging as described above. When indicated, digitonin-permeabilized Vero cells were preincubated with the polyvalent cationic agents at the concentrations described above for 2 h before performing import assays. For WGA treatment, samples were preincubated with WGA.

### Super Resolution-Structured Illumination Microscopy (SR-SIM)

After incubation of nuclei with GFP-labeled C-capsids (8×10^4^ counts of rat liver cell nuclei were incubated with approximately 10^7^ counts of capsids), the complete binding mixture was loaded onto chamber slides (Lab-Tek), and the samples were immediately imaged for GFP and DAPI using 405 nm and 488 nm excitation wavelengths with a Zeiss Elyra S1 microscope with a 64x-oil immersion lens. The images were captured on a sCMOS PCO Edge camera. The images were processed using the Structured Illumination module of the Zeiss (Zen 2011) software to obtain the super-resolved images of GFP-capsids bound to nuclei. The spatial resolution of the instrument is 120 nm. To generate 3D reconstructions, image stacks (1 μm) were acquired in Frame Fast mode with a z-step of 110 nm and 120 raw images per plane. Raw data were then computationally reconstructed using the Zen software to obtain a super-resolution 3D image stack. The Fiji-ImageJ software was used to generate a histogram of a cross-section profile for GFP-labeled C-capsid signal.

### Electron microscopy (EM)

After incubation of nuclei with C-capsids, the samples were washed once with CBB buffer (1,000 rpm for 10 min in a microcentrifuge). The supernatant was then removed and replaced with a fixative (2.5% EM-grade glutaraldehyde and 2.0% EM-grade formaldehyde in 0.1 M sodium cacodylate buffer, pH 7.4) for 3 hours at 4ºC. The fixative was then removed, replaced briefly with buffer, and then replaced with 1% osmium tetroxide in buffer for 90 minutes. Each sample was then subjected to 10-minute buffer rinse, after which it was placed in 1% aqueous uranyl acetate and left overnight. The next day, each sample was dehydrated via a graded ethanol series, culminating in propylene oxide. Following a graded propylene oxide; Epon812 series, the nuclear pellets were embedded in Epon812 prior to cutting. Ultrathin (ca. 90 nm) Epon sections on grids were stained with 1% aqueous uranyl acetate and lead citrate [98]. After the grids dried, areas of interest were imaged at 120 kV, spot 3 using a Tietz 2kx2k camera mounted on a Philips/FEI (now Thermo Fisher FEI) CM200 transmission electron microscope.

### In vitro viral replication assays conducted through NIH preclinical service agreement

**A**ssays were conducted in Dr. Mark Prichard’s laboratory, The University of Alabama at Birmingham, under NIH/NIAID preclinical service agreement. The protocols below were provided by Dr. Mark Prichard.

Each experiment that evaluated the antiviral activity of the compounds included both positive and negative control compounds to ensure the performance of each assay. Concurrent assessment of cytotoxicity was also performed for each study.

### Cell culture

Human foreskin fibroblast (HFF) cells prepared from human foreskin tissue were obtained from the University of Alabama at Birmingham tissue procurement facility with approval from its IRB. The tissue was incubated at 4°C for 4 h in Clinical Medium consisting of minimum essential media (MEM) with Earle’s salts supplemented with 10% fetal bovine serum (FBS) (Hyclone, Inc. Logan UT), l-glutamine, fungizone, and vancomycin. Tissue was then placed in phosphate-buffered saline (PBS), minced, rinsed to remove the red blood cells, and resuspended in trypsin/EDTA solution. The tissue suspension was incubated at 37°C and gently agitated to disperse the cells, which were collected by centrifugation. Cells were resuspended in 4 ml Clinical Medium, placed in a 25-cm^2^ flask, and incubated at 37°C in a humidified CO_2_ incubator for 24 h. The media was then replaced with fresh Clinical Medium, and the cell growth was monitored daily until a confluent monolayer had formed. The HFF cells were then expanded through serial passages in standard growth medium of MEM with Earle’s salts supplemented with 10% FBS, l-glutamine, penicillin, and gentamycin. The cells were passaged routinely and used for assays at or below passage 10 [99].

### Cytotoxicity assays

Cytotoxicity assays were performed on a parallel set of six-well plates containing HFF-1 cells that received the same compound concentrations as used for the antiviral assays but remained uninfected. The cytotoxicity plates were removed from the incubator on the same day as the cell monolayer was stained for 6 h with 2 ml of a neutral red solution at a concentration of 0.165 mg/ml in PBS. The residual dye was then removed by rinsing with PBS, and cell monolayers were inspected visually for any signs of toxicity.

### Neutral red uptake cytotoxicity assays

Each compound was evaluated in a standard cytotoxicity assay by standard methods [100]. Briefly, HFF-1 cells were seeded into 96-well tissue culture plates at a 2.5 x 10^4^ cells/well in standard growth medium. After 24 h of incubation, medium was replaced with MEM containing 2% FBS, and compounds were added to the first row and then 5-fold serial dilutes were used to generate a series of compound concentrations with a maximum of 300 μM. Assay plates were then incubated for 7 days, and 100 μl of a 0.66 mg/ml neutral red solution in PBS was added to each well, after which the plates were incubated for 1 h. The stain was then removed, the plates rinsed with PBS, and the dye internalized by viable cells was solubilized in PBS supplemented with 50% ethanol and 1% glacial acetic acid. The optical density was then determined at 550 nm and CC_50_ values were interpolated from the experimental data.

### Cell proliferation assays

The inhibition of either Vero cell or HFF-1 cell proliferation was used to refine estimates of cytotoxicity for the compounds and was performed according to standard procedures [101]. Cells were seeded at a low density into six-well plates using 2.5 x 10^4^ cells/well and standard culture medium. After 24 h, the medium was aspirated, and a range of compound solutions in the growth medium was prepared starting at 300 μM and added to duplicate wells. The plates were incubated for 72 h at 37°C; the cells were then dislodged with trypsin and counted on a Beckman Coulter Counter. Compound concentrations that reduced cell proliferation by 50% were interpolated from experimental data.

## Supporting information

S1 Text A. Control experiments for the reconstituted nuclei-capsid MOA-assay B. In vivo toxicity assay data obtained through an NIH/NIAID preclinical service agreement.
(PDF)

S1 Fig **SDS-PAGE verifying protein composition of purified A-, C-capsids and virions.** SDS-PAGE of A-capsid (lane 1), C-capsid (lane 2) and virions (lane 3) isolated from Vero cells infected with HSV-1 (KOS) (all in equivalent amounts). The proteins were detected by Coomassie blue staining. The positions of the HSV-1 virion proteins are shown on the right. Molecular mass standards are in kDa. Tegument, capsid and envelope proteins of the virions are indicated in the table. As expected, there was no difference in the protein profiles for A- and C- capsids, while tegument and envelope proteins were only detected for HSV-1 virions.
(PDF)

S2 Fig Effect of the compounds on the binding of HSV-1 virions to the cell surface during infection. (A). Binding of GFP-HSV-1 virions to the cell surface is blocked by the presence of bPEI, DAB-Am-4 or Arg^5+^. Confocal FM images of semiconfluent Vero-cell monolayers infected with GFP-expressing HSV-1 viruses at an MOI of 10. Cells were fixed at 2 hpi and assessed by the presence of a GFP virus signal (green). Nuclei were DAPI-stained (blue).
(PDF)

S3 Fig The integrity of isolated nuclei is not disrupted by the presence of external DNA condensing compounds. Isolated rat liver nuclei were incubated at 37ºC for 40 min in CBB buffer containing a fluorescently tagged 70 kDa dextran (green) and the compounds indicated. The nuclei were stained with DAPI (blue). DIC images of each field were also obtained (DIC panel). Complete dextran exclusion demonstrates the integrity of nuclei. Similar results were obtained in three independent experiments; a representative experiment is shown.
(PDF)

S4 Fig Control experiments showing that: 1. nuclear import of GFP-NLS-GST protein is not blocked by bPEI 600, DAB-Am-4, and Arg5+; 2. binding of GFP-HSV-1 C-capsids to nuclei of permeabilized cells is not blocked by bPEI, DAB-Am-4, and Arg5+. (A) Isolated rat liver nuclei were incubated with GST-NLS-GFP (green) at 37ºC for 30 min in CBB buffer containing rat liver cell cytosol and ATP regeneration system in the presence of DNA condensing compounds. As a negative control, we show that pre-incubation of nuclei with WGA prior to compound addition blocks NLS transport. (B) Vero cell monolayers permeabilized with digitonin were incubated with GST-NLS-EGFP (green) in the presence of tested compounds in A. The nuclei were stained with DAPI (blue). Control experiment shows that, in the absence of compounds, GST-NLS-GFP is fully transported into the nuclear interior. Addition of compounds does not interfere with this process. As a negative control, we show that pre-incubation of permeabilized cells with WGA prior to compound addition blocks NLS transport. (C) SR-SIM images of isolated GFP-HSV-1 capsids (green) bound to nuclei of Vero cells permeabilized with digitonin in the presence of different compounds at concentrations. Resolution is ~120 nm and individual capsids were observed. Selected compounds do not block capsid binding to nuclei. When permeabilized Vero cells were pretreated with 0.5 mg of WGA/mL, no capsid binding was observed, which illustrates binding specificity of nuclei to NPCs at the nuclear membrane.
(PDF)

S5 Fig Collection of higher magnification raw TEM images (25,000X) used to identify capsids that were associated with visible NPCs with and without different compounds added. The micrographs are from ultrathin-sectioned fixed/embedded samples. The ultrathin sections were stained with uranyl acetate and lead citrate. Prior to that, during the fixation process, the samples were immersed in 1% osmium tetroxide in buffer for 90 minutes.
(PDF)

S6 Fig Electron micrographs of HSV-1 C-capsids treated (lower panels) or untreated (upper panels) with bPEI for 1 hour. 250–300 capsids were analyzed and most representative images are shown. The top set of TEM images (C-capsids) is from negative-stained samples, stained with uranyl acetate. The bottom set of images (C-capsids+bPEI) is from ultrathin-sectioned fixed/embedded samples. The ultrathin sections were stained with uranyl acetate and lead citrate. Prior to that, during the fixation process, the samples were immersed in 1% osmium tetroxide in buffer for 90 minutes.
(PDF)

S7 Fig bPEI 25000 and DAB-Am-64 do not block binding of HSV-1 C-capsids to nuclei and do not interfere with nuclear transport functionality of NPCs. (A) Confocal fluorescence microscopy images show that binding of GFP-HSV-1 C-capsids (green) to DAPI-stained isolated nuclei (blue), in the presence of cytosol supplemented with an ATP-regeneration system, is not inhibited by addition of bPEI 25000 and DAB-Am-64. Addition of WGA prevents most of the capsid binding to nuclei, which demonstrates that capsids bind specifically to NPCs at the nuclear membrane. Bottom row 2 of the image shows a zoom-in version of an individual nucleus from images in row 1. (B) Isolated rat liver nuclei were incubated with GST-NLS-GFP (green) at 37ºC for 30 min in CBB buffer containing rat liver cell cytosol and ATP regeneration system in the presence of bPEI 25000 and DAB-Am-64. The compounds do not interfere with the nuclear transport through the NPCs. As a negative control, we show that pre-incubation of isolated nuclei with WGA prior to compound addition blocks NLS transport. (C) Vero cell monolayers permeabilized with digitonin were incubated with GST-NLS-EGFP (green) in the presence of tested compounds in C. The nuclei were stained with DAPI (blue). The compounds do not interfere with the nuclear transport through the NPCs. Control experiment shows that, in the absence of compounds, GST-NLS-GFP is fully transported into the nuclear interior. As a negative control, we show that pre-incubation of permeabilized cells with WGA prior to compound addition blocks NLS transport.
(PDF)

S1 Table DNA-DNA interaxial spacing *d*_*s*_-values for free DNA condensed by condensing compounds in TM-buffer, measured as a function of temperature. Vertical error bars are from the non-linear fitting of the DNA diffraction peak with a Gaussian function with background subtraction.
(PDF)

S2 Table DNA-DNA interaxial spacing *d*_*s*_-values for DNA packaged in HSV-1 C-capsid. Vertical error bars are from the non-linear fitting of the DNA diffraction peak with a Gaussian function with linear background subtraction.
(PDF)

S3 Table DNA-DNA interaxial spacing *d*_*s*_-values for DNA packaged in HSV-1 virion. Reduced *d*_*s*_-values show that compounds permeate the lipid envelope, tegument proteins and the capsid. The concentration of the polyvalent cationic agents was increased two and three times (2X, 3X) compared to the initial concentration in S1 Table. Virions were incubated with the compounds between 30 min and 12 hours (O.N., overnight.) prior to the measurement at 37°C.
(PDF)
