# Supplementary material for: Pressurized DNA state inside herpes capsids—A novel antiviral target
Source: PLoS Pathog. 2020 Jul 23;16(7):e1008604. doi: 10.1371/journal.ppat.1008604 (PMC7377361; doi:10.1371/journal.ppat.1008604)
Supplement: S1 Text — A. Control experiments for the reconstituted nuclei-capsid MOA-assay B. In vivo toxicity assay data obtained through an NIH/NIAID preclinical service agreement. (PDF) [file ppat.1008604.s001.pdf]

Supporting Information

Pressurized DNA state inside herpes capsids – A novel antiviral target

Alberto Brandariz-Nuñez[1], Scott J Robinson[2] and Alex Evilevitch[1,3,*]

1. Department of Pathobiology, College of Veterinary Medicine, University of Illinois at Urbana-Champaign, Urbana, IL 61802, USA

2. Beckman Institute for Advanced Science and Technology, University of Illinois at Urbana-Champaign, Urbana IL 61801, USA

3. Department of Experimental Medical Science, Lund University, BMC 113, SE-221 84 Lund, Sweden

**corresponding author:* alex.evilevitch@med.lu.se

A. Control experiments for the reconstituted nuclei-capsid MOA-assay (nuclear integrity, NPC transport functionality, digitonin-permeabilized cell assay). We first verified that the nuclear integrity and NPC transport function are unaffected by the presence of external DNA condensing compounds. Isolated intact nuclei exclude 70 kDa dextran fluorescently labeled with fluorescein isothiocyanate (FITC), which can be used to demonstrate their integrity. S3 Fig shows that the addition of selected DNA condensing compounds (DAB-Am-4, bPEI 600, and Arg^{5+}) did not alter the integrity of nuclei as shown by their ability to exclude FITC-labeled dextran. The sub-nuclear structure of heterochromatin DNA was also unchanged upon addition of these compounds (visualized by DAPI staining, S3 Fig). During infection, herpes DNA is ejected from capsids upon docking to the NPC and transported across the NPC channel into the nucleoplasm[1-3]. Therefore, we verified that NPC transport functionality is not disrupted by the presence of selected DNA condensing compounds. We performed an in vitro import assay to evaluate the nuclear import activity of NPCs using the nuclear localization signal (NLS)[4]. Purified rat liver nuclei were

incubated with cytosolic extracts (as a source of soluble import factors) supplemented with an ATP regeneration system and a purified GST-NLS-EGFP recombinant protein, which contains the nuclear localization signal (NLS) of the simian virus 40 T antigen fused with glutathione S-transferase (GST) and EGFP (see details in the *Materials and Methods* section). This protein was used as a positive import substrate, since it is transported into the nucleus by an active non-diffusion mechanism and can be detected by fluorescence microscopy[5, 6]. S4A Fig shows that the NPC maintained its full transport function of NLS in the presence of all three compounds. As a control we used WGA (wheat germ agglutinin), which blocks NPC receptor-mediated transport while not restricting passive diffusion (WGA associates with the glycoproteins within NPC[7, 8]). We confirmed that GST-NLS-EGFP was not transported across the nuclear membrane. To further demonstrate that selected compounds also do not block nuclear import through NPCs in cells, we used a digitonin-permeabilized cell assay (see *Materials and Methods* section), which accurately recapitulates nuclear import in living cells[9, 10]. In this standard import assay, adherent Vero cells were permeabilized with digitonin, and Vero cytosolic extracts (containing import factors) supplemented with an energy source and GST-NLS-EGFP were added to the permeabilized cells. In agreement with the previous observation in isolated nuclei, none of the selected compounds interfered with the nuclear import function of NPCs (S4B Fig). With addition of WGA, NLS was not transported into the nucleus and accumulated outside the nucleus.

Next, we confirmed that purified GFP-labelled HSV-1 DNA-filled C-capsids (strain K26GFP, HSV-1 strain expressing GFP-tagged VP26 protein) bind specifically to the NPC on isolated reconstituted nuclei and that binding is not inhibited by selected compounds. Cytosol was included in the nuclei-capsid mixture to improve capsid binding to nuclei[8, 11]. Super-Resolution Structured Illumination Microscopy (SR-SIM) in Fig 3A (*main text*), provides resolutions down to 120 nm[12], showing individual HSV-1 capsids (green labeled spots)–bound to NPC at the nuclear envelope boundary (see 2D and 3D images). Nuclei were stained with DAPI (blue). In parallel, confocal microscopy images in Fig 3B (*main text*) show that, by adding WGA and blocking NPCs[7], the capsid binding to nuclei was dramatically reduced, which confirms the presence of specific binding interactions between capsids and NPCs. We also show in Fig 3B that the presence of the selected DNA condensing compounds did not inhibit the binding of HSV-1 capsids to NPCs. In addition, we used a digitonin-permeabilized cell assay (described in the *Methods Section*), which reproduces capsids-nuclei binding in living cells[9, 10, 13]. Adherent

Vero cells were permeabilized with digitonin, and Vero cytosolic extracts along with purified GFP-labelled HSV-1 C-capsids were added to the permeabilized cells in the presence of compounds. Attachment of GFP-capsids to nuclei was visualized with SR-SIM (S4C Fig). In agreement with observations in the isolated nuclei system, we confirmed that none of the three compounds interfered with capsids-NPC binding in cells. WGA addition prevented capsid binding, showing capsid-NPC binding specificity (S4C Fig).

B. In vivo toxicity studies in mice for bPEI 600 and DAB-Am-4. Experiments were conducted through NIAID/NIH preclinical service agreement No HHSN272201700017I/ HHSN27200003.

Procedures and Endpoints:

1- Baseline weights of all mice
2- IP treatment (twice a day, for 4 days)
3- Daily assessment of weights and condition
4- All animals sacrificed the following morning after the last evening treatment
5- Blood samples collected by IC stick for lab tests (liver, spleen and blood profiles)
6- Internal organs examined for signs of gross tissue toxicity

1. Toxicity evaluation of bPEI 600 Da in mice.

A toxicity screen was performed in uninfected mice treated with 3 doses of bPEI 600 Da, administered for 4 days. In this study, 3 doses of 5, 10 and 25 mg/kg/day were administered twice daily IP for 4 days (n=4/group). A control group was treated with the vehicle (PBS). The mice were examined daily and the weights and general condition were recorded daily. To examine liver, renal and blood function parameters, blood samples were obtained by intracardiac puncture at the end of the study (the following day after last dose) and the mice were sacrificed to examine the internal organs for signs of gross tissue toxicity.

Results:

No abnormal signs were seen when the mice were evaluated daily. The weights were recorded daily and the results are shown in the following figure:

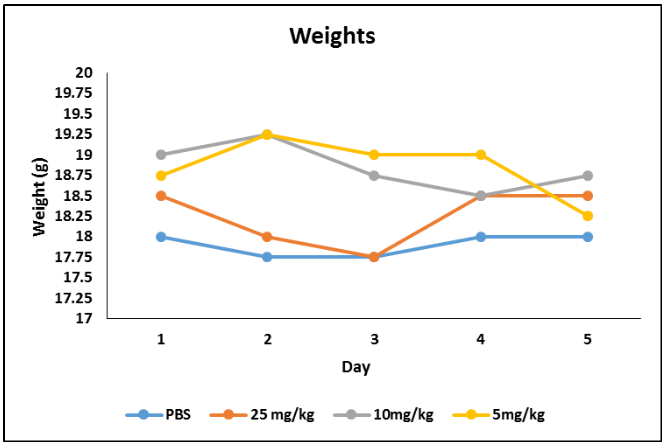

As seen in the figure above, the weight of the animals fluctuated ~ 1.0 g but no significant differences were found after 4 days of treatment. Blood samples were obtained for a comprehensive profile of renal, liver and blood functions. The mice in the study were slightly smaller than normally used for this type of the study, and the volume of blood necessary for all of the tests was difficult to obtain. The majority of the lab tests results were within normal parameters but an abnormal elevation in one of the liver enzymes (AST) was found in the treated groups ranging from a mean of 524 units/L in the 25 mg/kg/day dose to 1196 units/L in the 10 mg/kg/day dose. The control PBS group value was 240 units/L. Mild sample hemolysis may have occurred due to the difficulty experienced with some of the animals during the collection process, resulting in an elevation of the AST enzymes.

Mean Values - Renal, Liver, Blood Profiles

RENAL	PBS	5 mg/kg	10 mg/kg	25 mg/kg
Glucose	168	178	197	232
BUN	25	32	22	24
Uric Acid	6	---	2	2
Phosphorus	8	10	10	8
Calcium	8	7	8	7
Ca:Phos Ratio	1	1	4	1
Sodium	145	144	144	144
Potassium	7	10	10	10
Na: K Ratio	21	15	15	15
Chloride	109	111	110	109

LIVER	PBS	5 mg/kg	10 mg/kg	25 mg/kg
Total Protein	5	5	5	5
Albumin	3	3	3	3
Globin	2	2	2	2
Alb:Glob Ratio	2	1	2	2
**AST**	**240**	**715**	**1196**	**524**
GGT	<1	<1	1	2
Cholesterol	67	81	76	90
Triglyceride	156	160	163	146
Amylase	1374	833	702	938
Bile Acids	104	116	352	169
Creat Kin	**1000**	**2914**	**9447**	**-**

CBC	PBS	5 mg/kg	10 mg/kg	25 mg/kg
Hematocrit	41	42	40	44
WBC	9	6	8	9
%Lymphocyte	80	77	86	80

In order to confirm the results of the lab results obtained in the toxicity study, a second group of slightly larger mice were treated with the high and the low doses of bPEI 600 Da (25 mg/kg and 5 mg/kg, n=6/group) and the vehicle control (PBS, n=4) for 4 days. Blood samples were collected at the end of the study for liver and renal chemistry profiles. The results are shown in the next table:

Mean Values - Renal and Liver Profiles

RENAL	PBS	5 mg/kg	25 mg/kg
Glucose	186	256	211
BUN	29	25	34
Uric Acid	1	1	1
Phosphorus	9	8	8
Calcium	9	9	9
Ca:Phos Ratio	1	1	1
Sodium	151	150	153
Potassium	5	5	5
Na: K Ratio	34	32	34
Chloride	114	113	114

LIVER	PBS	5 mg/kg	25 mg/kg
Total Protein	5	4	5
Albumin	3	3	3
Globin	2	2	2
Alb:Glob Ratio	1	1	1
**AST**	257	187	211
GGT	<1	<1	<1
Cholesterol	78	69	69
Triglyceride	198	149	164
Amylase	836	690	1094
Bile Acids	1658	329	432
Creat Kin	45	247	88

Conclusion:

The results of the toxicity study indicated that treatment with bPEI 600 Da (25, 10 and 5 mg/kg, IP) was tolerated well. No signs of distress or significant weight loss were ere observed during the 4 days of treatment. The results of the lab profiles of the liver, renal and blood functions did not show significant differences with the results obtained in the PBS treated group of control mice.

Statistical analysis: Means were compared by Student's *t* test, and mortality was compared by Fisher's exact test, where applicable.

Ethics regulation of Laboratory animals: Animals were housed in facilities approved by the Association for Assessment and Accreditation of Laboratory Animal Care and Use Committee

2. Toxicity evaluation of DAB-Am-4 in mice.

A toxicity screen in uninfected mice was performed. 2 doses of 5 and 25 mg/kg/day were administered twice daily IP for 4 days (n=6/group). A control group was treated with the vehicle (PBS). The mice were examined daily and the weights and general condition were recorded. To examine liver, renal and blood function parameters, blood samples were obtained by intracardiac

puncture at the end of the study (the following day after last dose) and the mice were sacrificed to examine the internal organs for signs of gross tissue toxicity.

Results:

Measurements were performed for DAB-Am-4 and DAB-Am-8 compounds (both shown in the figure below). However, only compound DAB-Am-4 is tested in our manuscript since DAB-Am-8 displayed toxicity at highest concentration of 25 mg/kg/d. The weights were recorded daily and the results are shown in the following figure:

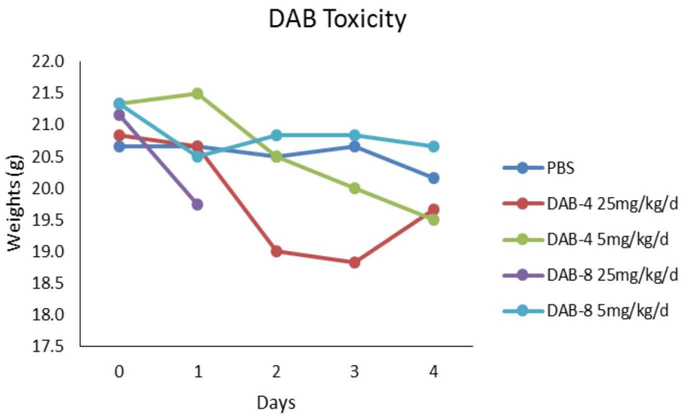

A seen in the figure above, the group treated with the high dose of 25 mg/kg/day DAB-Am-4 lost the most weight in the study (~10%, 2.0 g from baseline). The mice treated with the low dose (5 mg/kg/day) of both the DAB-Am-4 and DAB-Am-8 also lost weight, but no significant signs of distress were noted during the observation period.

After 4 days of treatment, blood samples were collected for renal, liver and CBC profiles. Emphasis was given to collecting samples for the renal and liver profiles, since these parameters would be more likely to show signs of acute toxicity. The results of the lab tests performed are summarized in the following figure:

	PBS		DAB-Am-4 25 mg/kg/day		DAB-Am-4 5 mg/kg/day		DAB-Am-8 5 mg/kg/day	
	Mean	STDEV	Mean	STDEV	Mean	STDEV	Mean	STDEV
**RENAL (n=5)**								
Glucose	233	45	182	20	221	36	204	23
BUN	27	3	36	6	35	4	22	3
Uric Acid	3	2	5	1	3	1	2	0
Phosphorus	9	1	9	2	11	2	9	1
Calcium	8	0	9	0	9	1	8	0
Ca:Phos Ratio	1	0	1	0	1	0	1	0
Na	147	1	147	1	143	5	147	3
K	5	1	6	0	7	2	7	1
Na: K Ratio	29	4	25	1	22	6	22	4
Chloride	113	2	106	1	104	4	111	2
**LIVER (n=5)**								
Total Protein	4	0	5	0	5	0	4	0
Albumin	3	0	3	0	3	0	3	0
Globin	2	0	2	0	2	0	2	0
Alb:Glob Ratio	2	0	1	0	1	0	2	0
AST	224	144	414	180	402	144	437	482
GGT	<1		<1		<1		<1	
Cholesterol	65	3	85	3	91	15	61	5
Triglyceride	147	26	149	17	118	29	114	18
Amylase	771	159	760	96	792	128	730	128
Creatine Kinase	2184	1770	3089	1551	2340	1026	3149	4249
Bile Acids	10	5	13	21	134	275	238	294
**CBC (n=1-2)**								
Hematocrit	42		36		43	0	42	
WBC	6		4		7	3	6	
%Lymphocyte	80		61		67	5	75	

As seen in the results above, no conclusive signs of acute toxicity were found in the groups treated with 5 and 25 mg/kg/day of DAB-Am-4 and the low dose (5 mg/kg/day) of DAB-Am-8.

Conclusion:

The DAB-Am-4 treated mice showed weigh loss but appeared to recover on the last day of observation (25 mg/kg/day).

Statistical analysis: Means were compared by Student's *t* test, and mortality was compared by Fisher's exact test, where applicable.

Ethic regulation of Laboratory animals: Animals were housed in facilities approved by the Association for Assessment and Accreditation of Laboratory Animal Care and Use Committee.

References:

1. Fay N, Panté NJFim. Nuclear entry of DNA viruses. 2015;6:467.
2. Kobiler O, Drayman N, Butin-Israeli V, Oppenheim AJN. Virus strategies for passing the nuclear envelope barrier. 2012;3(6):526-39.
3. Mettenleiter TCJJomb. Breaching the barrier—The nuclear envelope in virus infection. 2016;428(10):1949-61.
4. Miyamoto Y, Hieda M, Harreman MT, Fukumoto M, Saiwaki T, Hodel AE, et al. Importin alpha can migrate into the nucleus in an importin beta- and Ran-independent manner. EMBO J.

2002;21(21):5833-42. Epub 2002/11/02. PubMed PMID: 12411501; PubMed Central PMCID: PMCPMC131066.
5. Tsuji T, Sheehy N, Gautier VW, Hayakawa H, Sawa H, Hall WWJJoBC. The nuclear import of the human T lymphotropic virus type I (HTLV-1) tax protein is carrier-and energy-independent. 2007;282(18):13875-83.
6. Vázquez-Iglesias L, Lostalé-Seijo I, Martínez-Costas J, Benavente JJJov. Avian reovirus sigmaA localizes to the nucleolus and enters the nucleus by a nonclassical energy-and carrier-independent pathway. 2009;83(19):10163-75.
7. Finlay DR, Newmeyer DD, Price TM, Forbes DJJTJocb. Inhibition of in vitro nuclear transport by a lectin that binds to nuclear pores. 1987;104(2):189-200.
8. Ojala PM, Sodeik B, Ebersold MW, Kutay U, Helenius A. Herpes simplex virus type 1 entry into host cells: reconstitution of capsid binding and uncoating at the nuclear pore complex in vitro. Mol Cell Biol. 2000;20(13):4922-31. PubMed PMID: 10848617.
9. Adam SA, Marr RS, Gerace LJTJoCB. Nuclear protein import in permeabilized mammalian cells requires soluble cytoplasmic factors. 1990;111(3):807-16.
10. Cassany A, Gerace L. Reconstitution of nuclear import in permeabilized cells. The Nucleus: Springer; 2008. p. 181-205.
11. Anderson F, Savulescu AF, Rudolph K, Schipke J, Cohen I, Ibiricu I, et al. Targeting of viral capsids to nuclear pores in a cell-free reconstitution system. 2014;15(11):1266-81.
12. Sekine E, Schmidt N, Gaboriau D, O'Hare P. Spatiotemporal dynamics of HSV genome nuclear entry and compaction state transitions using bioorthogonal chemistry and super-resolution microscopy. PLoS Pathog. 2017;13(11):e1006721. Epub 2017/11/10. doi: 10.1371/journal.ppat.1006721. PubMed PMID: 29121649; PubMed Central PMCID: PMCPMC5697887.
13. Au S, Wu W, Zhou L, Theilmann DA, Panté N. A novel mechanism for nuclear import by actin-based propulsion used by the baculovirus nucleocapsid. J Cell Sci. 2016:jcs. 191668.
