# Supplementary material for: Pressurized DNA state inside herpes capsids—A novel antiviral target
Source: PLoS Pathog. 2020 Jul 23;16(7):e1008604. doi: 10.1371/journal.ppat.1008604 (PMC7377361; doi:10.1371/journal.ppat.1008604)
Supplement: S1 Fig — SDS-PAGE verifying protein composition of purified A-, C-capsids and virions. SDS-PAGE of A-capsid (lane 1), C-capsid (lane 2) and virions (lane 3) isolated from Vero cells infected with HSV-1 (KOS) (all in equivalent amounts). The proteins were detected by Coomassie blue staining. The positions of the HSV-1 virion proteins are shown on the right. Molecular mass standards are in kDa. Tegument, capsid and envelope proteins of the virions are indicated in the table. As expected, there was no difference in the protein profiles for A- and C- capsids, while tegument and envelope proteins were only detected for HSV-1 virions. (PDF) [file ppat.1008604.s002.pdf]

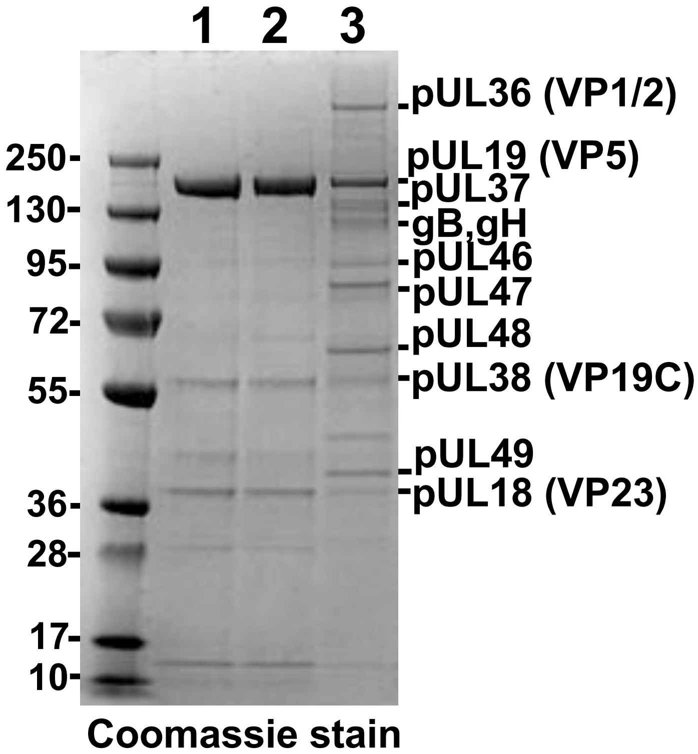

Tegument proteins	Capsid proteins	Envelope proteins
UL36 (VP1/2)<br>UL37	UL19 (VP5)<br>UL38 (VP19C)	gB
UL46, UL47,<br>UL48 UL49	UL18 (VP3)	gH

Fig. S1
