# Supplementary figures and images for: Pressurized DNA state inside herpes capsids—A novel antiviral target
Source: PLoS Pathog. 2020 Jul 23;16(7):e1008604. doi: 10.1371/journal.ppat.1008604 (PMC7377361; doi:10.1371/journal.ppat.1008604)

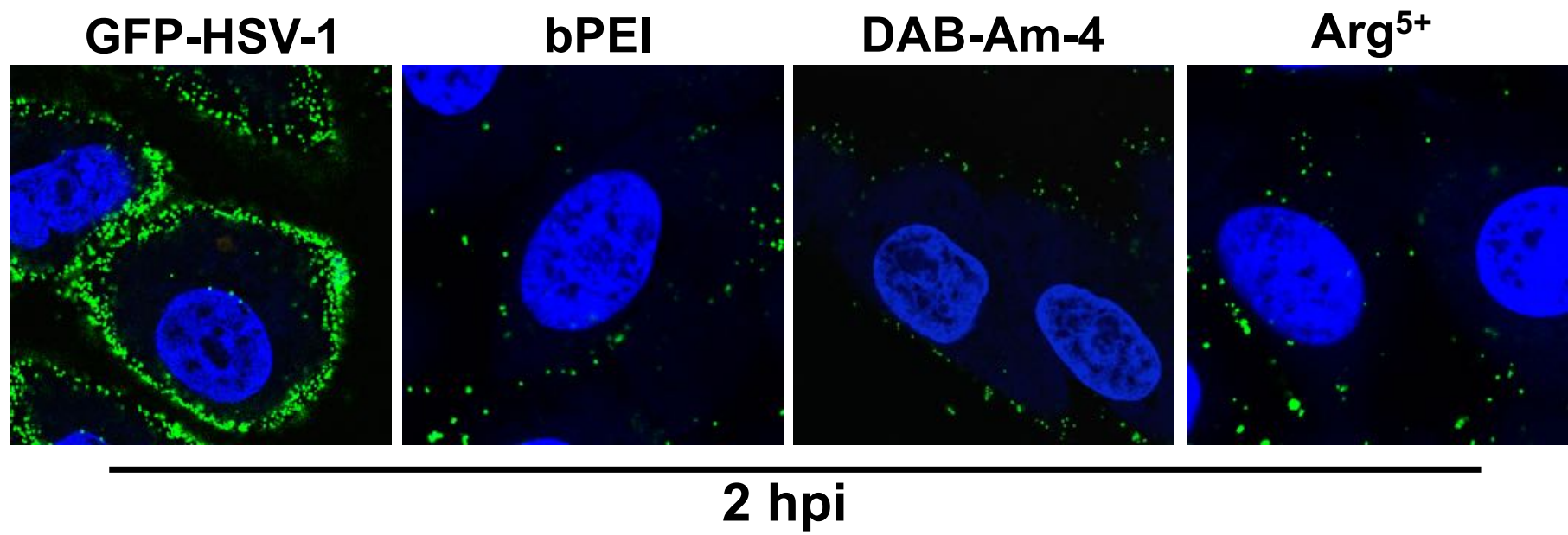

**Fig. S2**

Supplement: S2 Fig — (A). Binding of GFP-HSV-1 virions to the cell surface is blocked by the presence of bPEI, DAB-Am-4 or Arg5+. Confocal FM images of semiconfluent Vero-cell monolayers infected with GFP-expressing HSV-1 viruses at an MOI of 10. Cells were fixed at 2 hpi and assessed by the presence of a GFP virus signal (green). Nuclei were DAPI-stained (blue). (PDF) [file ppat.1008604.s003.pdf]

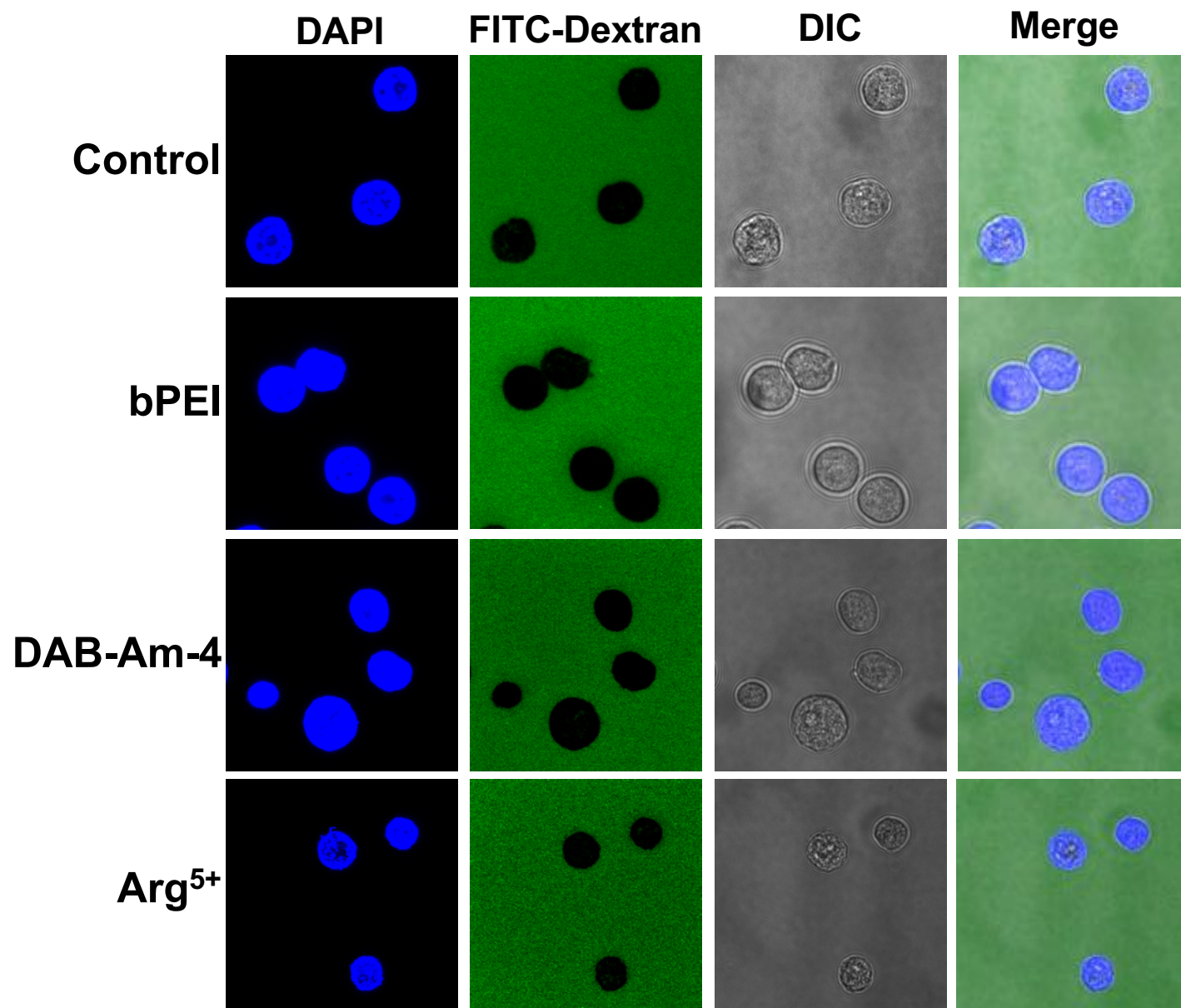

**Fig. S3**

Supplement: S3 Fig — Isolated rat liver nuclei were incubated at 37ºC for 40 min in CBB buffer containing a fluorescently tagged 70 kDa dextran (green) and the compounds indicated. The nuclei were stained with DAPI (blue). DIC images of each field were also obtained (DIC panel). Complete dextran exclusion demonstrates the integrity of nuclei. Similar results were obtained in three independent experiments; a representative experiment is shown. (PDF) [file ppat.1008604.s004.pdf]

**A**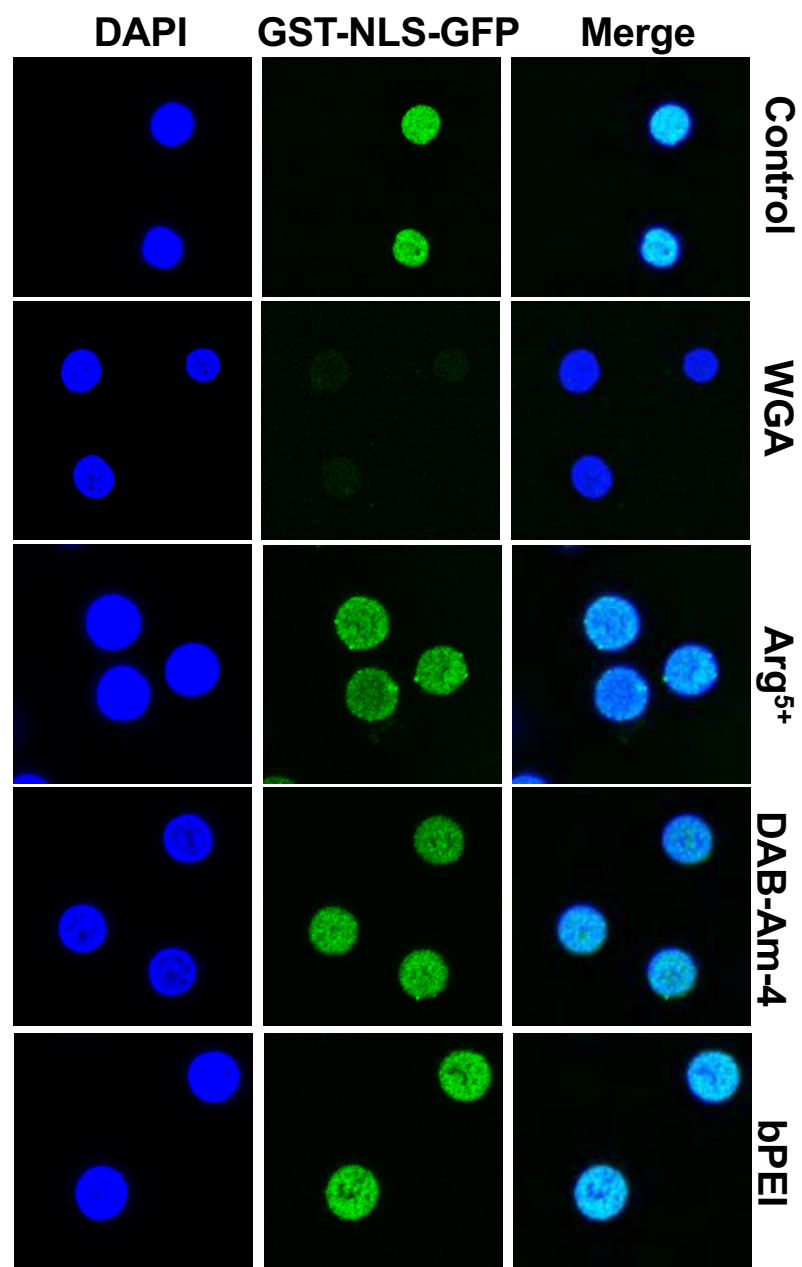**B**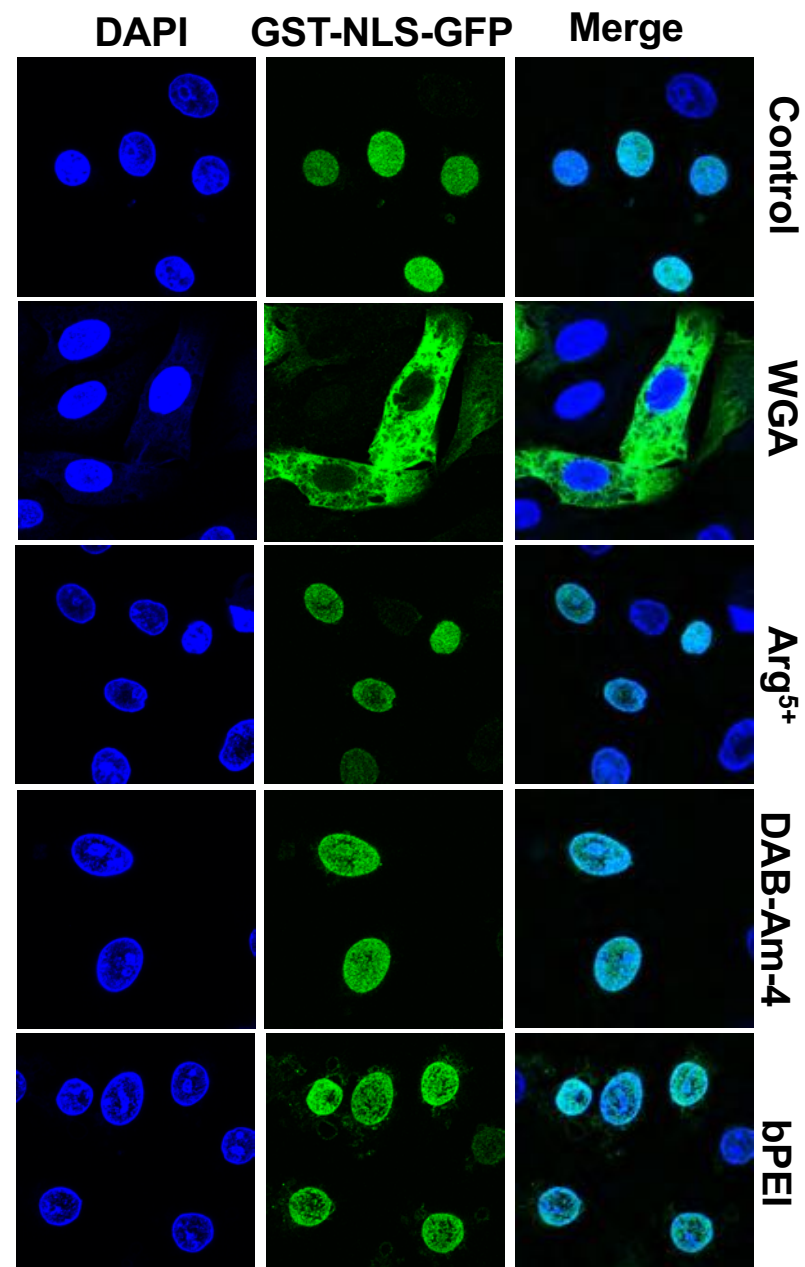**Fig. S4 A and B**

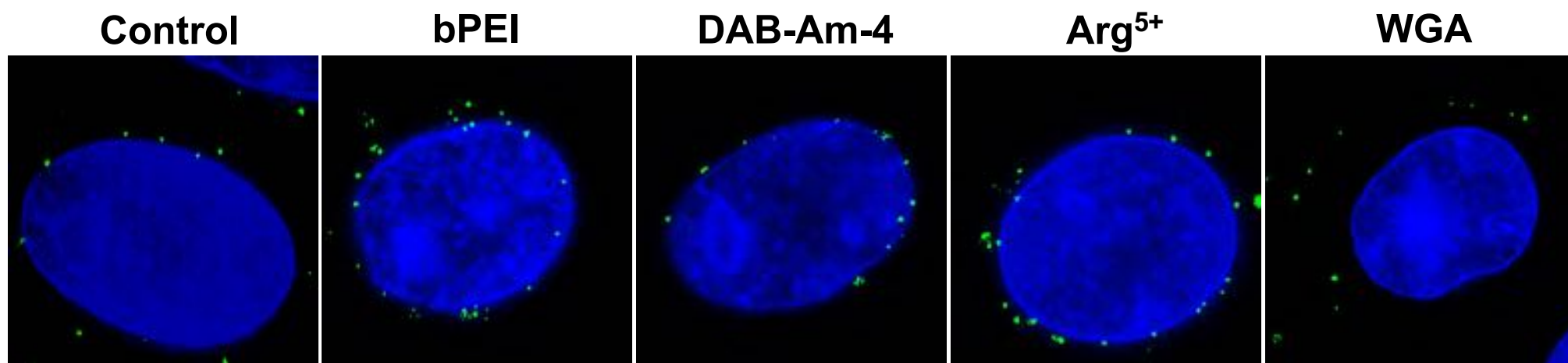

**Fig. S4 C**

Supplement: S4 Fig — (A) Isolated rat liver nuclei were incubated with GST-NLS-GFP (green) at 37ºC for 30 min in CBB buffer containing rat liver cell cytosol and ATP regeneration system in the presence of DNA condensing compounds. As a negative control, we show that pre-incubation of nuclei with WGA prior to compound addition blocks NLS transport. (B) Vero cell monolayers permeabilized with digitonin were incubated with GST-NLS-EGFP (green) in the presence of tested compounds in A. The nuclei were stained with DAPI (blue). Control experiment shows that, in the absence of compounds, GST-NLS-GFP is fully transported into the nuclear interior. Addition of compounds does not interfere with this process. As a negative control, we show that pre-incubation of permeabilized cells with WGA prior to compound addition blocks NLS transport. (C) SR-SIM images of isolated GFP-HSV-1 capsids (green) bound to nuclei of Vero cells permeabilized with digitonin in the presence of different compounds at concentrations. Resolution is ~120 nm and individual capsids were observed. Selected compounds do not block capsid binding to nuclei. When permeabilized Vero cells were pretreated with 0.5 mg of WGA/mL, no capsid binding was observed, which illustrates binding specificity of nuclei to NPCs at the nuclear membrane. (PDF) [file ppat.1008604.s005.pdf]

**C-capsids**

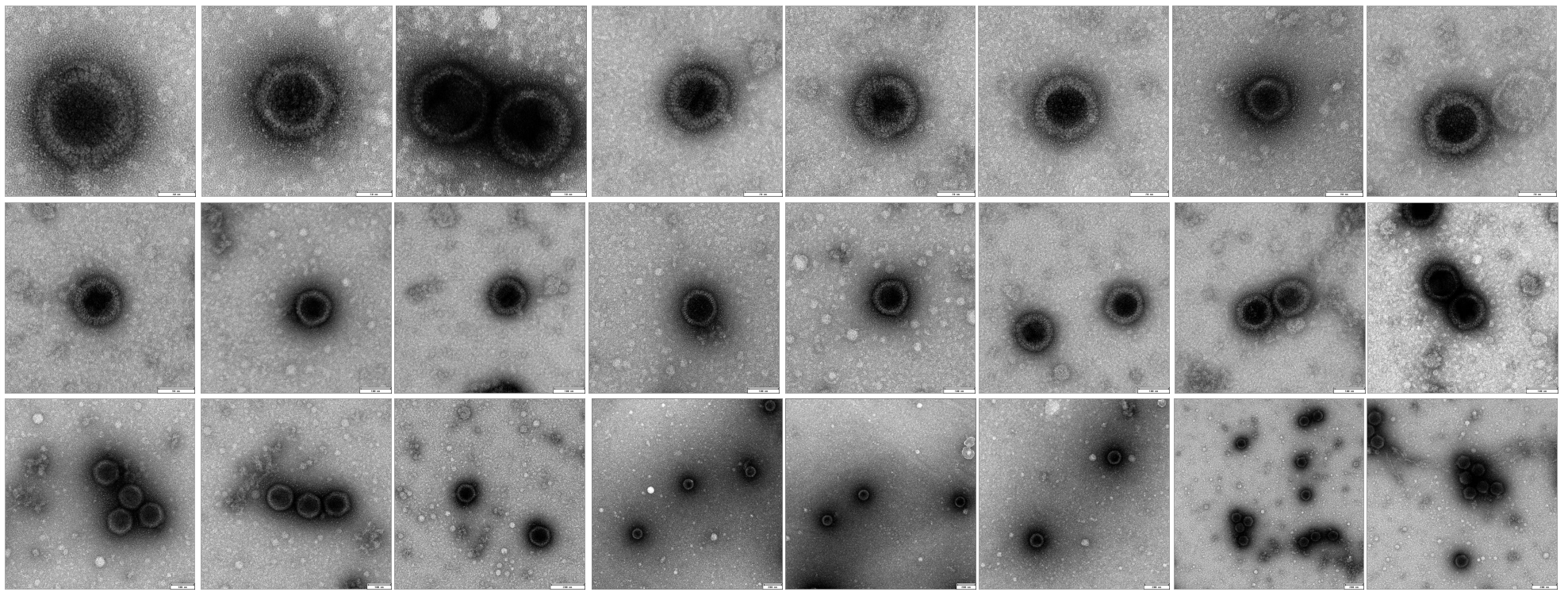

**C-capsids+bPEI**

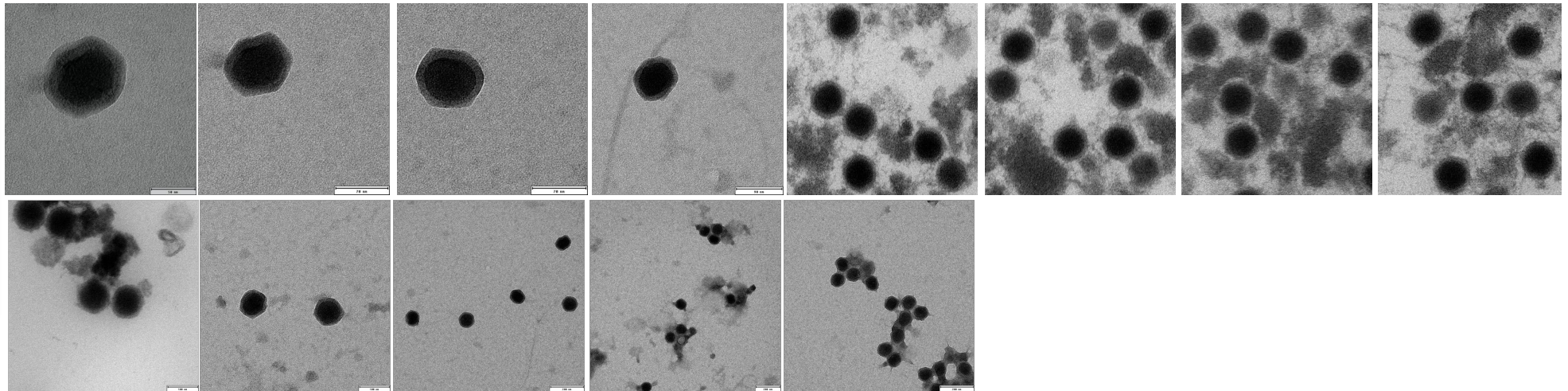

**Fig. S6**

Supplement: S6 Fig — 250–300 capsids were analyzed and most representative images are shown. The top set of TEM images (C-capsids) is from negative-stained samples, stained with uranyl acetate. The bottom set of images (C-capsids+bPEI) is from ultrathin-sectioned fixed/embedded samples. The ultrathin sections were stained with uranyl acetate and lead citrate. Prior to that, during the fixation process, the samples were immersed in 1% osmium tetroxide in buffer for 90 minutes. (PDF) [file ppat.1008604.s007.pdf]

**A**

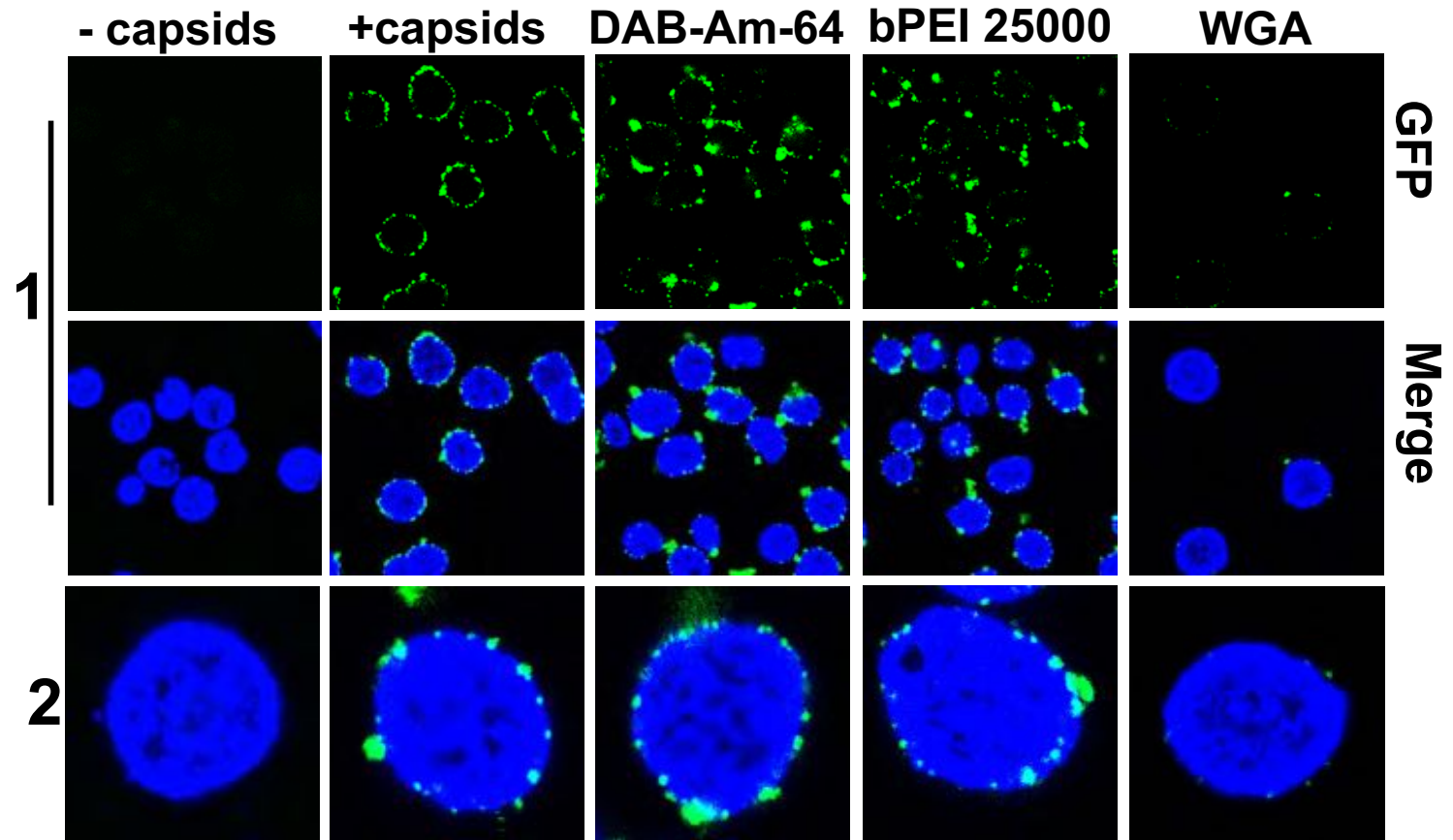

**Fig. S7A**

**B**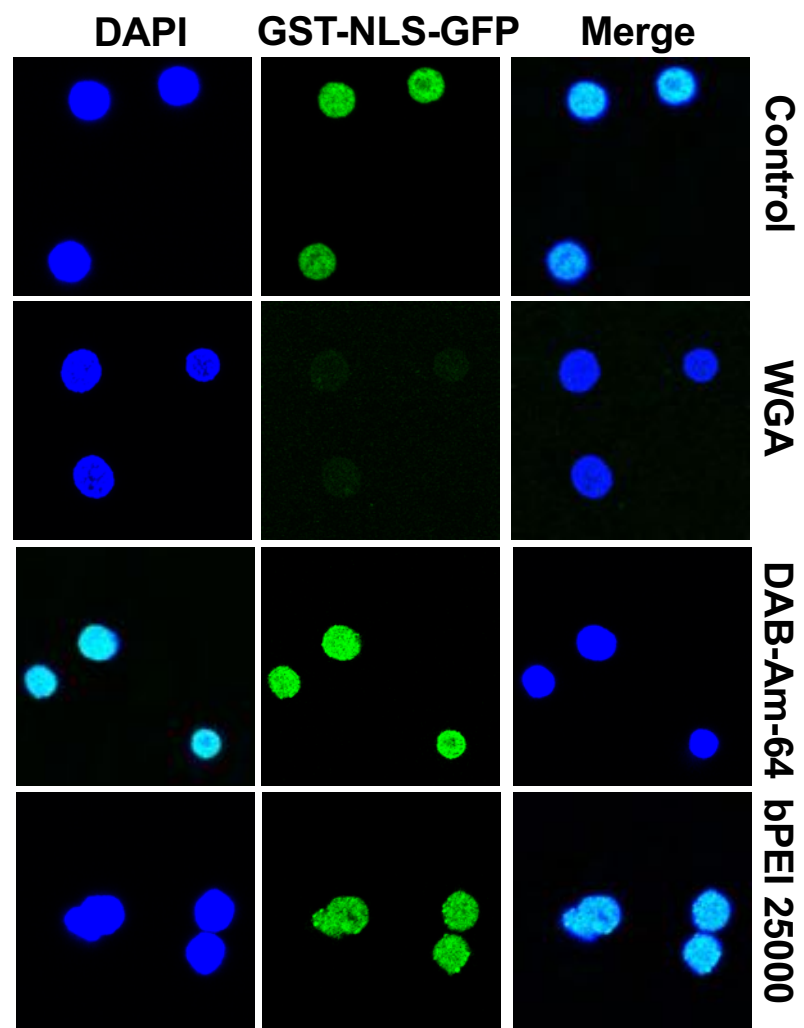**C**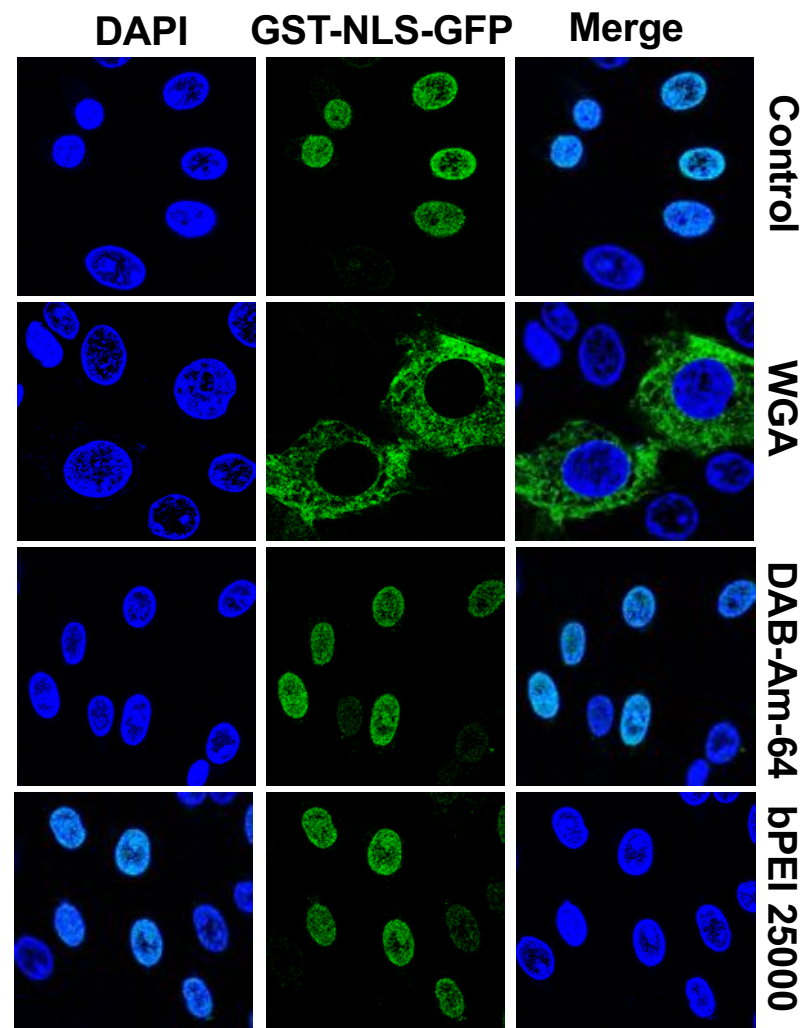

**Figs. S7 B & C**

Supplement: S7 Fig — (A) Confocal fluorescence microscopy images show that binding of GFP-HSV-1 C-capsids (green) to DAPI-stained isolated nuclei (blue), in the presence of cytosol supplemented with an ATP-regeneration system, is not inhibited by addition of bPEI 25000 and DAB-Am-64. Addition of WGA prevents most of the capsid binding to nuclei, which demonstrates that capsids bind specifically to NPCs at the nuclear membrane. Bottom row 2 of the image shows a zoom-in version of an individual nucleus from images in row 1. (B) Isolated rat liver nuclei were incubated with GST-NLS-GFP (green) at 37ºC for 30 min in CBB buffer containing rat liver cell cytosol and ATP regeneration system in the presence of bPEI 25000 and DAB-Am-64. The compounds do not interfere with the nuclear transport through the NPCs. As a negative control, we show that pre-incubation of isolated nuclei with WGA prior to compound addition blocks NLS transport. (C) Vero cell monolayers permeabilized with digitonin were incubated with GST-NLS-EGFP (green) in the presence of tested compounds in C. The nuclei were stained with DAPI (blue). The compounds do not interfere with the nuclear transport through the NPCs. Control experiment shows that, in the absence of compounds, GST-NLS-GFP is fully transported into the nuclear interior. As a negative control, we show that pre-incubation of permeabilized cells with WGA prior to compound addition blocks NLS transport. (PDF) [file ppat.1008604.s008.pdf]
