# Supplementary material for: Pressurized DNA state inside herpes capsids—A novel antiviral target
Source: PLoS Pathog. 2020 Jul 23;16(7):e1008604. doi: 10.1371/journal.ppat.1008604 (PMC7377361; doi:10.1371/journal.ppat.1008604)
Supplement: S5 Fig — The micrographs are from ultrathin-sectioned fixed/embedded samples. The ultrathin sections were stained with uranyl acetate and lead citrate. Prior to that, during the fixation process, the samples were immersed in 1% osmium tetroxide in buffer for 90 minutes. (PDF) [file ppat.1008604.s006.pdf]

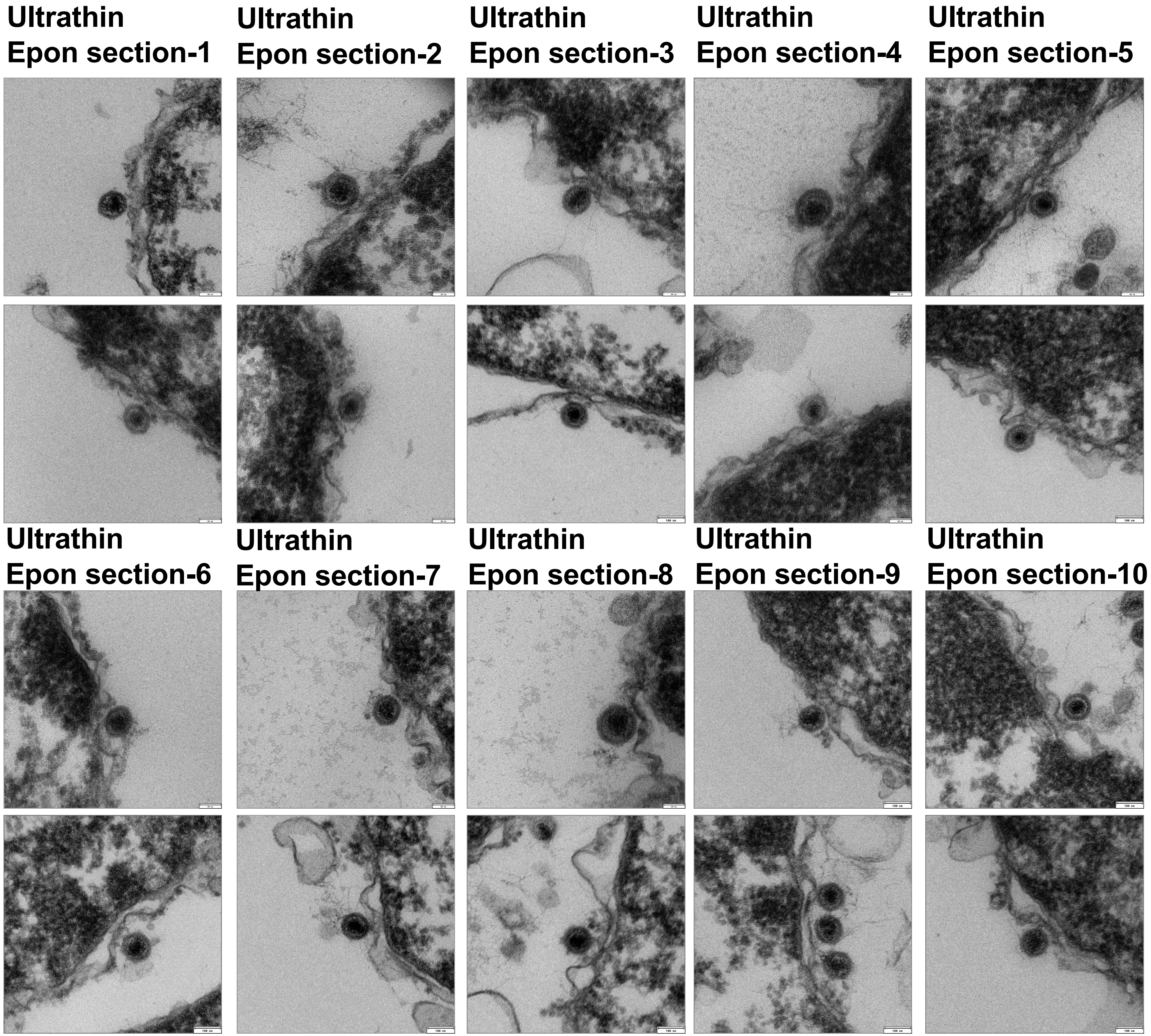

Ultra-thin sectioning EM shows capsids in the absence of compounds (capsids were mixed with nuclei supplemented with cytosol). Negative control at 4°C, without added compounds and without ATP regenerating system, shows that no ejection occurs.

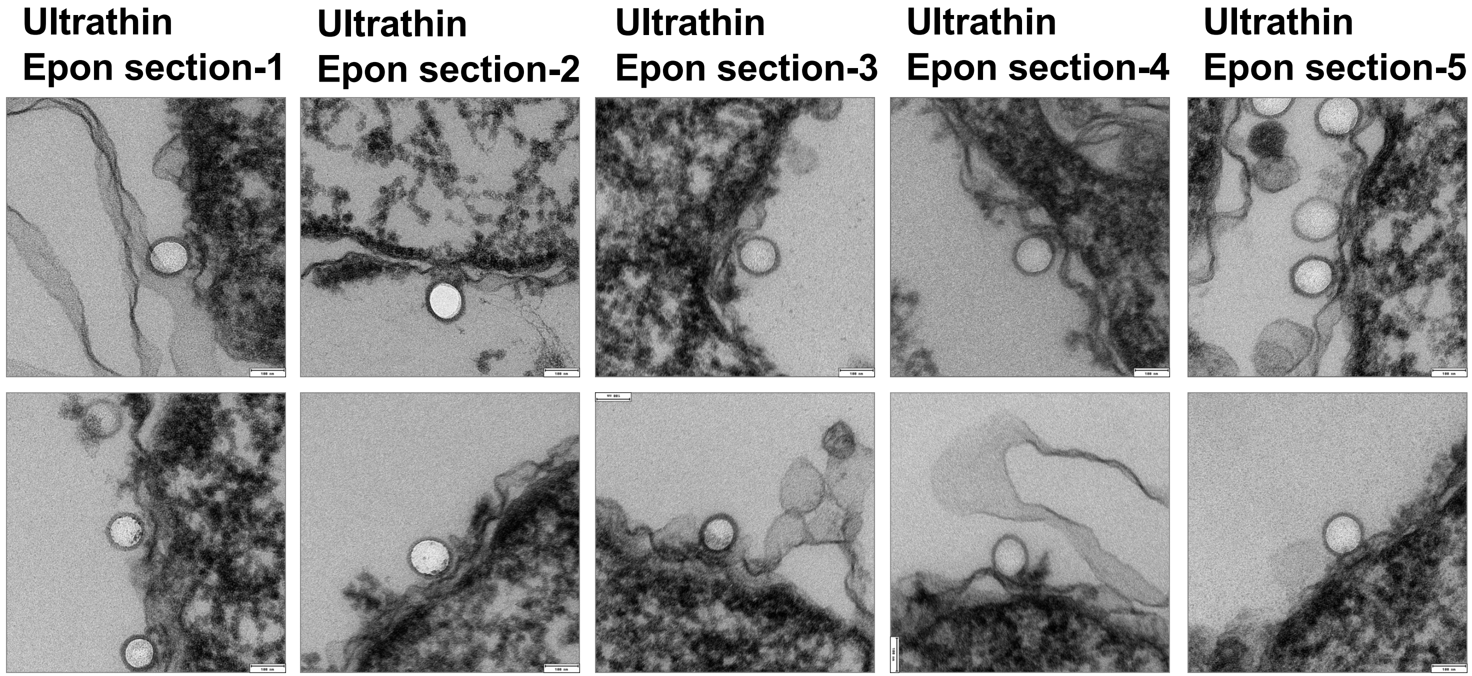

Ultra-thin sectioning EM shows capsids in absence of compounds. Positive control at 37°C shows complete DNA ejection from C-capsids in the absence of compounds (capsids were mixed with nuclei supplemented with cytosol and ATP-regenerating system).

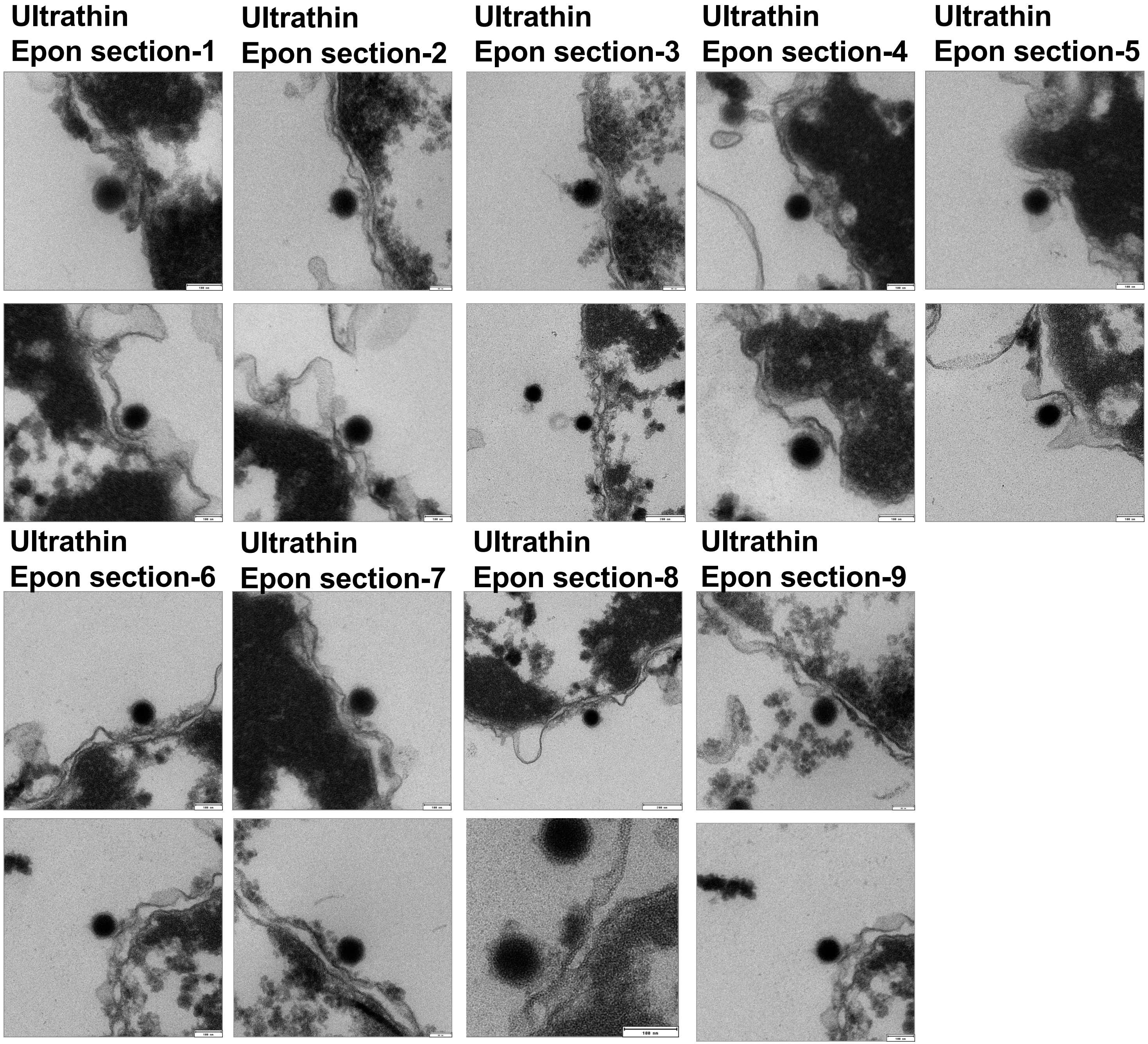

Ultra-thin sectioning EM shows that the addition of the selected DNA condensing compound (bPEI 600) inhibits DNA ejection from HSV-1 C-capsids into a cell nucleus through the NPCs.

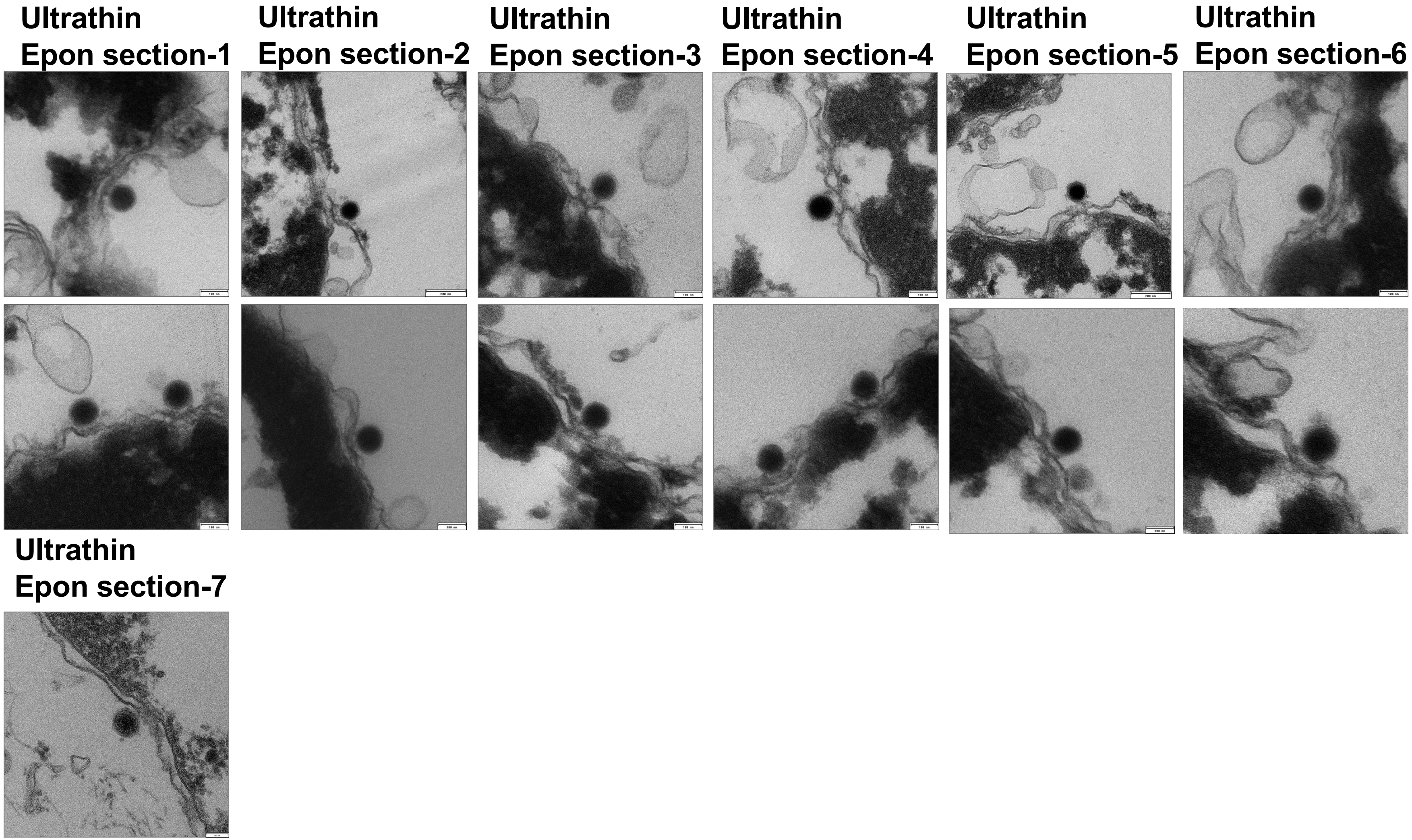

Ultra-thin sectioning EM shows that the addition of the selected DNA condensing compound (DAB-Am-4) inhibits DNA ejection from HSV-1 C-capsids into a cell nucleus through the NPCs.

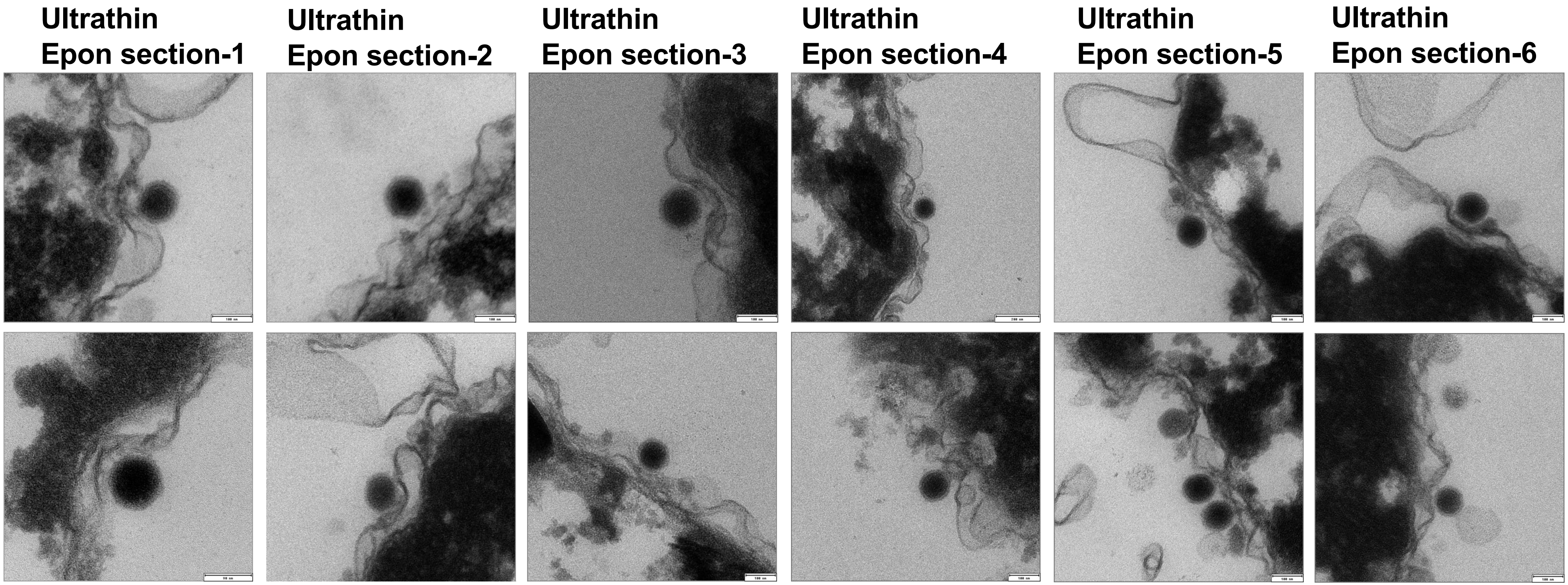

Ultra-thin sectioning EM shows that the addition of the selected DNA condensing compound (Arg5+) inhibits DNA ejection from HSV-1 C-capsids into a cell nucleus through the NPCs.

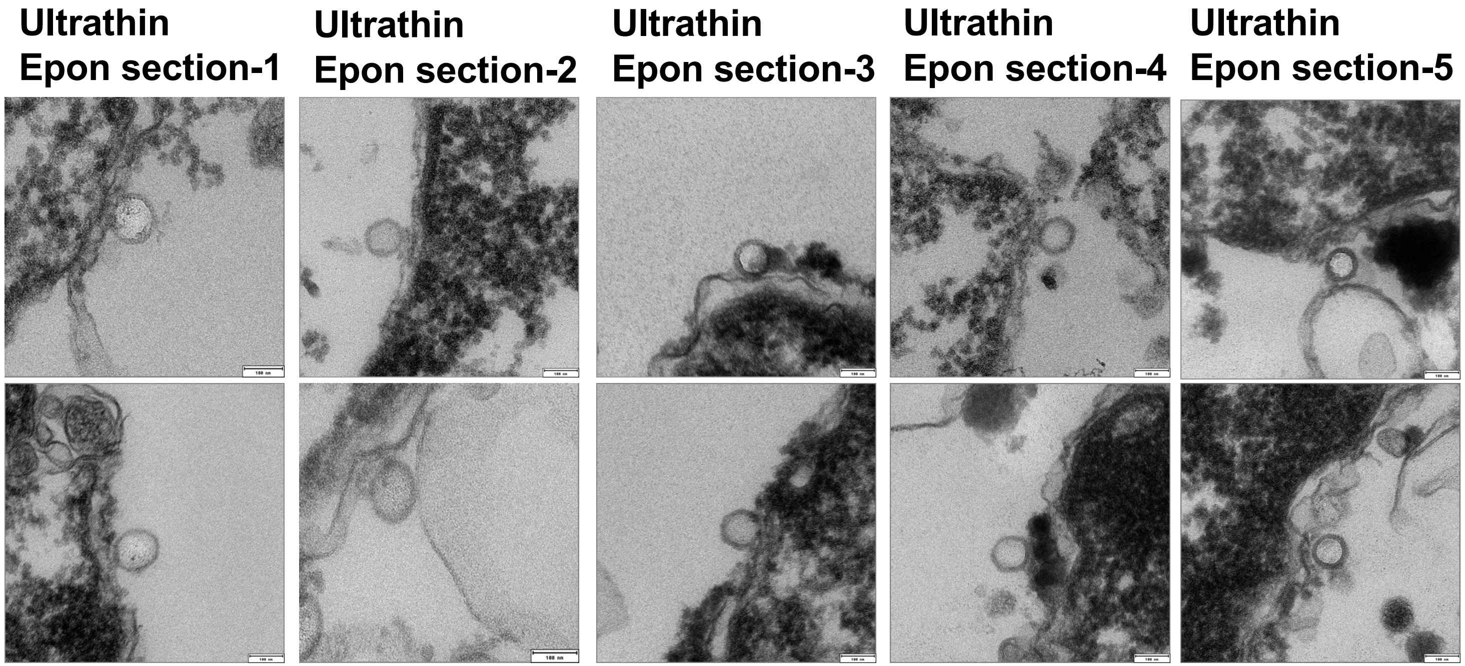

Ultra-thin sectioning EM shows that addition of bPEI 25000 does not inhibit DNA ejection from HSV-1 C-capsids into a cell nucleus through the NPC.

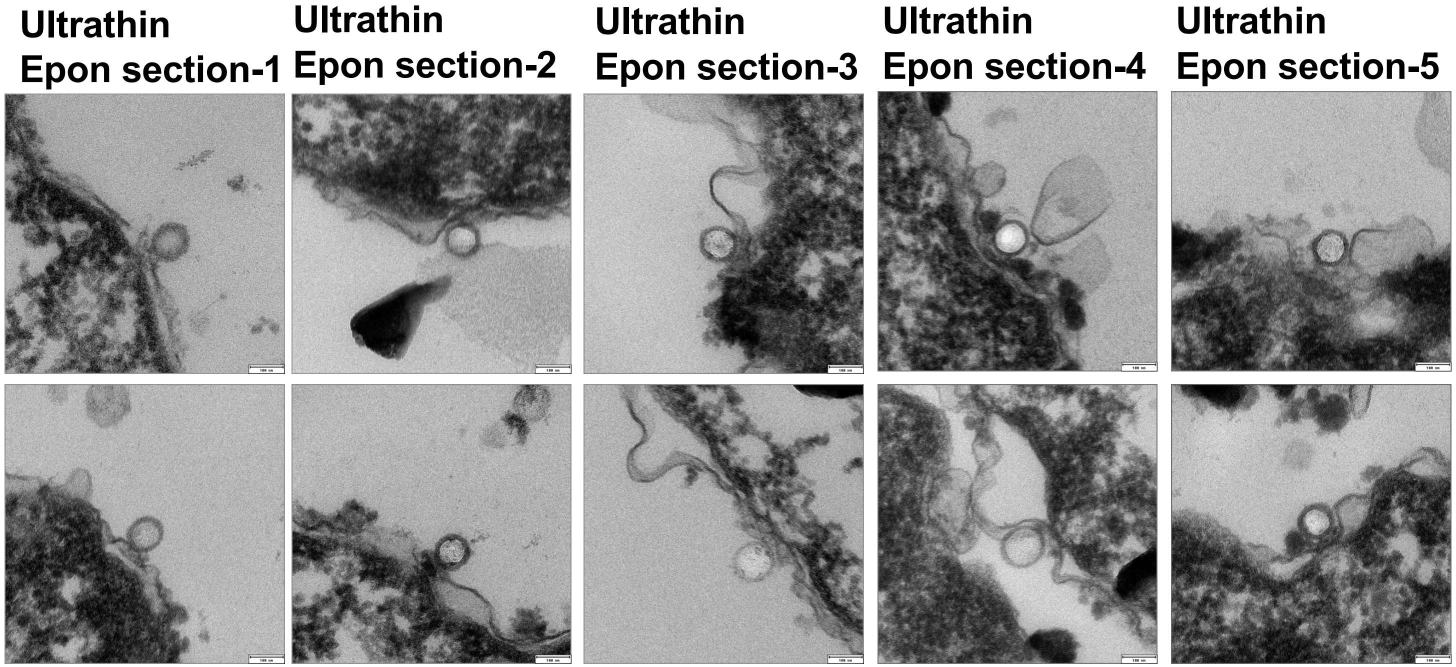

Ultra-thin sectioning EM shows that addition of DAB-Am-64 does not inhibit DNA ejection from HSV-1 C-capsids into a cell nucleus through the NPC.
