# Supplementary material for: Pressurized DNA state inside herpes capsids—A novel antiviral target
Source: PLoS Pathog. 2020 Jul 23;16(7):e1008604. doi: 10.1371/journal.ppat.1008604 (PMC7377361; doi:10.1371/journal.ppat.1008604)
Supplement: S1 Table — Vertical error bars are from the non-linear fitting of the DNA diffraction peak with a Gaussian function with background subtraction. (PDF) [file ppat.1008604.s009.pdf]

Compound	DNA-DNA d-spacing (Å)		
	Temperature (ºC)		
	15ºC	22ºC	37ºC
**Arg^{5+}**	29.26 ± 0.01	29.39 ± 0.07	29.21 ± 0.05
**bPEI**	28.20 ± 0.01	28.22 ± 0.01	28.19 ± 0.01
**DAB-Am-4**	29.08 ± 0.01	29.04 ± 0.01	28.55 ± 0.01

Table S1
