# Supplementary material for: Pressurized DNA state inside herpes capsids—A novel antiviral target
Source: PLoS Pathog. 2020 Jul 23;16(7):e1008604. doi: 10.1371/journal.ppat.1008604 (PMC7377361; doi:10.1371/journal.ppat.1008604)
Supplement: S2 Table — Vertical error bars are from the non-linear fitting of the DNA diffraction peak with a Gaussian function with linear background subtraction. (PDF) [file ppat.1008604.s010.pdf]

	DNA-DNA d-spacing (Å)		
	Temperature (ºC)		
**Compound**	**15ºC**	**22ºC**	**37ºC**
**C-capsid**	**31.31 ± 0.08**	**31.16 ± 0.07**	**31.02 ± 0.12**
**Arg^{5+}**	29.71 ± 0.06	29.78 ± 0.05	29.86 ± 0.06
**bPEI**	29.48 ± 0.04	29.39 ± 0.04	29.18 ± 0.04
**DAB-Am-4**	29.78 ± 0.04	29.73 ± 0.04	29.47 ± 0.05

Table S2
