# Supplementary material for: Pressurized DNA state inside herpes capsids—A novel antiviral target
Source: PLoS Pathog. 2020 Jul 23;16(7):e1008604. doi: 10.1371/journal.ppat.1008604 (PMC7377361; doi:10.1371/journal.ppat.1008604)
Supplement: S3 Table — Reduced ds-values show that compounds permeate the lipid envelope, tegument proteins and the capsid. The concentration of the polyvalent cationic agents was increased two and three times (2X, 3X) compared to the initial concentration in S1 Table. Virions were incubated with the compounds between 30 min and 12 hours (O.N., overnight.) prior to the measurement at 37°C. (PDF) [file ppat.1008604.s011.pdf]

DNA-DNA d-spacing (Å)

Compound	Concentration			
	1X	2X	2X	3X
**HSV-1 virion**	**31.03 ± 0.10**	**30.90 ± 0.12**	**31.00 ± 0.06**	**30.99 ± 0.06**
**Arg^{5+}**	30.39 ± 0.09	30.27 ± 0.11	30.22 ± 0.06	30.23 ± 0.05
**bPEI**	30.36 ± 0.09	30.51 ± 0.08	30.50 ± 0.09	30.46 ± 0.11
**DAB-Am-4**	30.18 ± 0.08	30.42 ± 0.09	30.52 ± 0.09	30.52 ± 0.10
			**O.N. incubation**	

Table S3
